# Necroptosis triggers inflammatory interferon signatures in patient-derived metastatic breast cancer organoids

**DOI:** 10.1038/s41392-026-02755-9

**Published:** 2026-06-01

**Authors:** Kaja Nicole Wächtershäuser, Jana V. Schneider, Alec Gessner, Geoffroy Andrieux, Ivan-Maximiliano Kur, Nadine Duschek, Andreas Weigert, Melanie Boerries, Michael A. Rieger, Ernst H. K. Stelzer, Francesco Pampaloni, Sjoerd J. L. van Wijk

**Affiliations:** 1 Buchmann Institute for Molecular Life Sciences, Faculty of Biological Sciences (IZN), Goethe University Frankfurt am Main, Frankfurt am Main, Germany; 2 Department of Medicine II, Hematology/Oncology, Goethe University Frankfurt am Main, Frankfurt am Main, Germany; 3 Institute of Medical Bioinformatics and Systems Medicine, Medical Center – University of Freiburg, Faculty of Medicine, University of Freiburg, Freiburg, Germany; 4 Department for Immunity of Inflammation, Mannheim Institute for Innate Immunoscience (MI3), Medical Faculty Mannheim, Heidelberg University, Mannheim, Germany; 5 Institute for Experimental Pediatric Hematology and Oncology, Goethe University Frankfurt am Main, Frankfurt am Main, Germany; 6 Institute of Biochemistry I, Faculty of Medicine, Goethe University Frankfurt am Main, Frankfurt am Main, Germany; 7 German Cancer Consortium (DKTK) partner site Frankfurt/Mainz and German Cancer Research Center (DKFZ), Heidelberg, Germany; 8 Frankfurt Cancer Institute (FCI), Frankfurt am Main, Germany; 9 German Cancer Consortium (DKTK), Partner site Freiburg, a partnership between DKFZ and Medical Center - University of Freiburg, Freiburg, Germany; 10 University Cancer Centre Frankfurt (UCT), University Hospital Frankfurt, Goethe University Frankfurt am Main, Frankfurt am Main, Germany; 11 Institute for Physiology and Cell Biology, University of Veterinary Medicine, Hannover Foundation, Hannover, Germany

**Keywords:** Breast cancer, Cancer models, Cancer stem cells

## Abstract

Breast cancer (BC) is the most common type of cancer among women worldwide and underlies relapse, disease progression, and metastasis. Resistance to chemotherapy and programmed cell death (PCD), including apoptosis, strongly affects BC therapy success and remains a major challenge. Although necroptosis, a lytic, and via damage-associated molecular patterns (DAMP) release, highly immunogenic mode of PCD, might overcome apoptosis resistance, there is an urgent need for physiological and translational human models to model necroptosis and PCD resistance in BC. Here, we apply 3D patient-derived, metastatic human mammary organoids (hMOs) to model apoptosis resistance, necroptosis, and inflammatory signaling using single-cell CITE-sequencing, time-lapse live cell brightfield, and immunofluorescent confocal microscopy, as well as biochemical approaches. Smac mimetic-triggered apoptosis could be confirmed in a panel of BC hMOs. Upon inducing apoptosis resistance with caspase inhibition, BC hMOs rapidly undergo necroptosis with profound MLKL phosphorylation. Necroptotic cell death was preceded by prominent transcriptional upregulation of inflammatory cyto- and chemokines, including interferons, that activate natural killer cells. Finally, necroptosis and the expression and release of inflammatory messengers in metastatic BC hMOs could be attenuated upon the inhibition of linear ubiquitination. We describe a novel experimental platform to model PCD, inflammation, and necroptosis that allows therapeutic screening to overcome chemotherapy resistance in patient-derived metastatic BC hMOs. With this platform, we identified necroptosis-induced interferon signaling, suggesting Smac mimetics and necroptosis as a potential immunotherapy strategy against metastatic BC.

## Introduction

Breast cancer (BC) is the most commonly diagnosed type of cancer among women worldwide, leading to high numbers of cancer-related deaths in females with steadily increasing incidence.^1,2^ The heterogeneity of BC, coupled with a rapid adaptation of tumor cells – involving metastasis and the development of resistance to chemotherapeutics and programmed cell death (PCD) mechanisms – significantly worsens the prognosis and overall disease outcome.^2,3^ Adequate experimental models to investigate and predict treatment responses, and at the same time recapitulate the complex near-physiological multicellular situation in human BC tissues, remain sparse. Adult stem cell-derived organoids represent clinically and physiologically relevant experimental systems to study chemotherapy-treated as well as metastatic BC in vitro and allow the investigation of multicellular tumor growth, PCD mechanisms, and preclinical drug testing.^4,5^ These organoid cultures are established from patient tissues and maintain the characteristics of the original tumors^6^ and can be expanded from primary tumors, metastatic lesions, and healthy tumor-adjacent tissues.^6–8^ Organoids are grown in three-dimensional (3D) matrices that mimic the extracellular matrix (ECM)^9^ and possess an inherent heterogeneity.^10^ Patient-derived BC human mammary organoids (hMOs) reflect the clinical response of patients prior to treatment and predict drug sensitivities^4^ and allow personalized drug screening to overcome chemotherapy and PCD resistance in highly patient- and clinically relevant settings^11^ in line with the Food and Drug Administration (FDA) Modernization Act 2.0.^12^ Therefore, patient-derived organoids are bridging the gap between in vitro and in vivo studies.^13^

Resistance to apoptosis often stems from altered expression of the cellular inhibitor of apoptosis protein (cIAP) as well as X-linked IAP (XIAP) E3 ligases that are typically highly expressed in a wide variety of cancers.^14,15^ Second mitochondria-derived activator of caspases (Smac) is the natural antagonist of IAPs and induces protein clustering, leading to IAP auto-ubiquitination and proteasomal degradation.^16^ A promising strategy to induce PCD in IAP-overexpressing tumors is by treatment with Smac mimetics, including TL32711 (Birinapant), BV6, LCL-161, and ASTX660, and this strategy has reached clinical phase I and II studies in various hematological and solid tumors.^17,18^ Mechanistically, cIAPs, as well as XIAP, typically mediate NF-ĸB, JNK, and p38-dependent pro-survival responses via ubiquitination of cellular targets, including the receptor-interacting serine/threonine-protein kinase 1 (RIPK1), often downstream of death receptors, such as TNFR1.^19,20^ Loss of RIPK1 ubiquitination upon IAP inhibition triggers the formation of pro-apoptotic complexes that lead to caspase cleavage and the execution of apoptosis.^21,22^ Importantly, resistance to apoptosis may be acquired through mutations in extrinsic apoptosis pathways or via the overexpression of pro-survival proteins during intrinsic apoptosis.^23^ Therefore, the induction of alternative PCD pathways might be evaluated as alternative targets for cancer treatment. Caspase-independent forms of PCD, such as necroptosis, can overcome apoptosis resistance. Necroptosis is characterized by the auto- and trans-phosphorylation of RIPK1 and RIPK3, leading to the recruitment and phosphorylation of the mixed lineage kinase domain-like pseudokinase (MLKL).^24^ Phosphorylated MLKL oligomerizes and forms pores in the plasma membrane, allowing the release of cytokines, chemokines, and damage-associated molecular patterns (DAMPs).^25^ Therefore, necroptosis can circumvent apoptosis resistance and trigger inflammatory responses in the cellular microenvironment to support anti-tumor immune responses.^25,26^

Linear ubiquitination, in which ubiquitin molecules are connected via the initiator methionine of ubiquitin instead of lysine residues, is an important checkpoint for cell survival.^27^ Linear ubiquitin is catalyzed by the E3 ubiquitin ligase linear ubiquitin chain assembly complex (LUBAC), and both linear ubiquitin and LUBAC play important roles in inflammatory signaling, including NF-κB, downstream of cytokine receptor signaling, PCD control, and innate immunity.^28–30^ Genetic deregulation of LUBAC function and linear ubiquitin signaling underly tumorigenesis in lymphoma, lung squamous cell carcinoma, leukemias, and breast cancer^27^ and inflammatory disorders.^31–36^ Inhibition of HOIP, the catalytic subunit of LUBAC, with the small molecule HOIPIN-8 mimics genetic LUBAC loss of function and suppresses NF-κB signaling and modulates cell viability.^37^ HOIPIN-8 acts by covalently binding to the catalytic residue C885 of HOIP and by interfering with the linear ubiquitin chain determining domain (LDD) within HOIP.^37^ We have recently demonstrated that HOIPIN-8 also attenuates and delays the onset of necroptosis in human cancer cell lines and pancreatic organoids^38^ by preventing membrane accumulation of MLKL.

To investigate apoptosis resistance and alternative forms of PCD in physiological near-patient multicellular BC settings, we describe a translational experimental pipeline based on primary hMOs derived from metastatic lesions of BC patients. This setup allows for organoid- and single-cell-specific monitoring, modeling, and therapeutic manipulation of apoptosis resistance, necroptosis, and linear ubiquitination. By applying this approach, we show efficient monitoring and therapeutic modulation of apoptosis and necroptosis and demonstrate prominent interferon responses during Smac mimetic-induced necroptosis, contributing to natural killer cell activation and anti-tumor immune regulatory functions in BC hMOs. Taken together, this study provides a proof-of-principle experimental framework to study the mechanisms, responses, and sensitivities of PCD in primary BC hMOs and allows the future development, application, and evaluation of (semi-throughput) drug screening for personalized and immune cell-based treatment strategies.

## Results

### Establishing a translational pipeline to characterize PCD and inflammation in metastatic BC hMOs

To bridge the gaps between in vitro and in vivo BC models and to minimize the use of animal models, we investigated how patient-derived organoids can be applied as preclinical models to study multicellular tumor growth and sensitivities towards PCD. We utilized advanced, metastatic BC hMOs, that maintained the original tumor characteristics and that were derived from adult stem cells and obtained from primary patient tissues.^6,7^ Using these patient-derived hMOs, we aimed to set up an experimental system to systematically model PCD sensitivity and inflammation. Metastatic BC is poorly understood, mostly due to its heterogeneity, and presents a challenge because of resistance to the most common treatments.^39^ For this, three hMOs lines from metastatic lesions were obtained from biobanking, and stable cultures were established in a chemically defined medium that mimicked the tumor microenvironment. Donor #1 was initially diagnosed with luminal B BC, whereas donors #2 and #3 presented luminal A BC.^6,7^ Patient-derived hMO growth and expansion were monitored optically using brightfield, immunofluorescence and confocal microscopy.

**Fig. 1:**
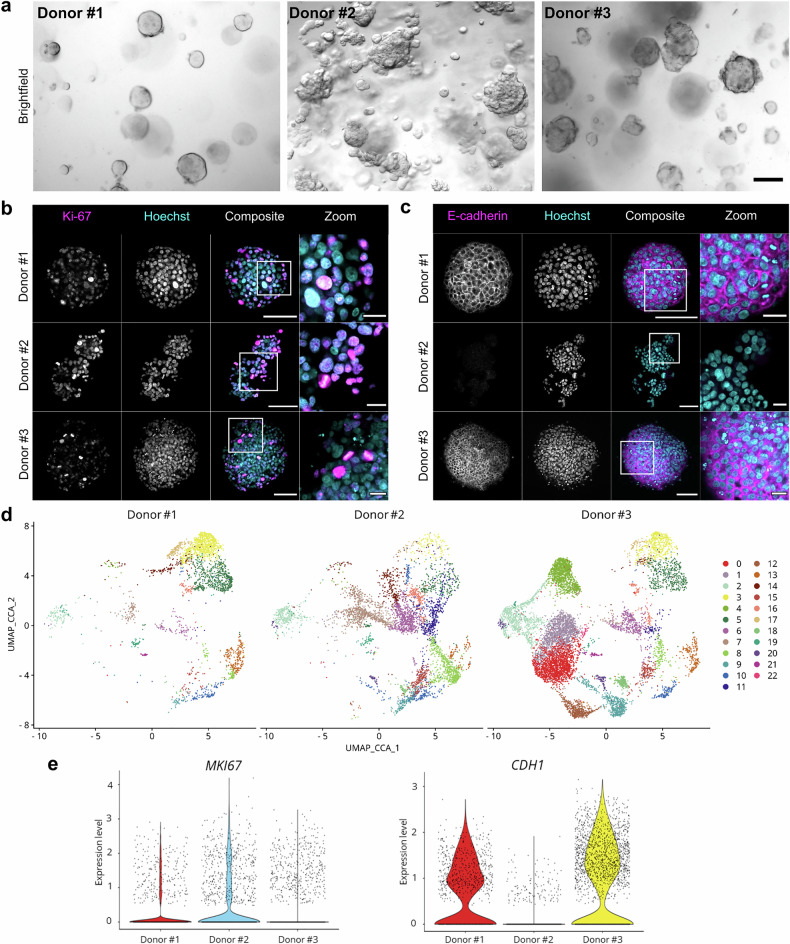
Establishing a translational pipeline to characterize PCD and inflammation in primary BC hMOs. **a** hMOs were imaged after organoid formation in brightfield microscopy using the Zeiss SteREO Discovery.V8 microscope. Scale bar: 250 µm. hMOs were fixed and subjected to immunofluorescent staining against Ki-67 (**b**, magenta) or E-cadherin (**c**, magenta) and counterstained using Hoechst33342 (cyan). hMOs were cleared with CUBIC-2 and imaged using a Zeiss LSM780 confocal microscope. Scale bar: 100 µm, enlarged image: 25 µm. **d** Control-treated hMOs (1.5% DMSO for 24 h) were subjected to scCITEseq. All data were combined, and uniform manifold approximation and projection (UMAP) analysis was performed. **e** Violin plots of *MKI67* and *CDH1* expression levels from scCITEseq data

Consistent with the clinical situation,^6^ the three hMO lines exhibited distinct morphological features (Fig. 1a) with different expansion rates during routine culturing (Supplementary Fig. 1a). hMOs derived from donor #1 and donor #3 presented a compact, round phenotype, whereas the BC organoids of donor #2 proliferated in a “grape-like” morphology. In accordance with their luminal origin, the three organoid lines expressed cytokeratin-8, a luminal cell marker, but lacked the expression of cytokeratin-14, a basal cell marker (Supplementary Fig. 1b, c). All hMOs contained Ki67-positive nuclei, but more Ki67-positive cells could be detected in the less dense growing hMOs from donor #2 (Fig. 1b), indicating that Ki67 levels reflected expansion rates. hMOs from donors #1 and #3 expressed E-cadherin, whereas no E-cadherin could be detected in hMOs from donor #2 (Fig. 1c), suggesting a potential link between E-cadherin expression and proliferation.^40,41^

hMOs from all three donors were subjected to single-cell CITE-Seq (scCITEseq) and the data were divided into 22 clusters following principal component analysis (Fig. 1d and Supplementary Fig. 2a). Cell numbers per condition and donor were listed in Supplementary Fig. 2b, and all conditions per donor were combined and processed individually to identify clusters (Supplementary Fig. 2c). Analysis of *CDH1* and *MKI67* mRNA expression, encoding E-cadherin and Ki67, showed that hMOs from donor #3 expressed the lowest amounts of *MKI67*, whereas donor #2 almost completely lacked *CDH1* expression, confirming expression levels observed in immunofluorescence staining (Fig. 1e).

### Metastatic hMOs express luminal BC markers with donor-specific expression signatures

**Fig. 2:**
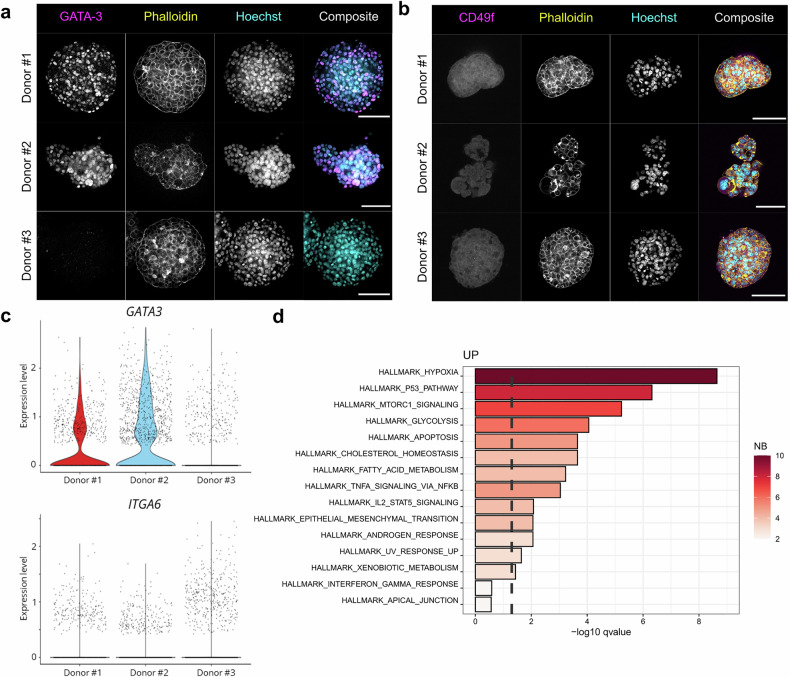
Metastatic hMOs exhibit a luminal like BC subtype with donor-specific expression signatures. hMOs were fixed prior to immunofluorescence staining against GATA-3 (**a**, magenta) or CD49f (**b**, magenta) and counterstained with AF647™-Phalloidin (yellow) and Hoechst33342 (cyan). CUBIC-2 cleared hMOs were imaged with a Zeiss LSM780 confocal microscope. Scale bar: 100 µm. **c** Violin plots of *GATA3* and *ITGA6* expression from scCITEseq data confirmed immunofluorescence data. **d** scCITEseq was subjected to gene set enrichment analysis (GSEA) using the hallmark dataset. Control-treated hMOs (1.5% DMSO) from all donors were combined, and the most upregulated signaling pathways were shown. The x-axis represents the statistical significance of enrichment (–log₁₀ adjusted q-value), and the y-axis lists the top enriched gene sets. Bars are color-coded according to the number of differentially expressed genes (DEGs) contained within each gene set. The dashed vertical line indicates the threshold for statistical significance (-log_10_ adjusted q-value(0.05))

To further investigate donor-specific differences in mammary marker expression, GATA-3 and CD49f expression was evaluated using immunofluorescence and confocal microscopy (Fig. 2a, b). GATA-3 is a marker for luminal cells^42^ and is highly expressed in luminal A BC.^43^ The highest levels of GATA-3 expression were detected in hMOs from donor #2, and to a lower extent in donor #1 (Fig. 2a), and showed nuclear localization. Donor #3 exhibited almost no GATA3-positive cells. Analysis of scCITEseq data confirmed the lack of *GATA3* expression in donor #3 (Fig. 2c). CD49f, also referred to as integrin-α6 and encoded by *ITGA6*, is a biomarker for breast cancer^44^ and was detected in hMOs from all three donors (Fig. 2b), although scCITEseq revealed low mRNA expression levels (Fig. 2c). Lastly, gene set enrichment analysis (GSEA) was performed using the hallmark dataset.^45,46^ First, the datasets from the three donors were combined to investigate commonalities between the donors and to identify trends in gene expression profiles. The combined hallmark analysis revealed strong induction of hypoxia signaling as well as the p53 pathway (Fig. 2d). Especially hypoxia has been linked to BC progression through activation of glycolysis, angiogenesis, and metastasis^47^ in line with the metastatic origin of the organoids.

To evaluate donor-specific differences between the hMOs, pseudo-bulk GSEA was applied on the combined dataset of all donors, after which the most upregulated hallmarks between the three different donors were compared. MYC signaling was the most characteristic hallmark for donor #1, whereas donor #2 showed more hypoxic signaling, glycolysis, and TNF-α signaling via NF-κB. Last, hMOs from donor #3 showed high estrogen response in concordance with the media comprising β-estradiol (Supplementary Fig. 3a–c). Analyzing the donor-specific individual datasets revealed that hMOs from donors #1 and #3, both exhibiting the compact organoid morphology, display strong MTORC1 signaling, which is commonly associated with worse BC prognosis and is considered to be oncogenic.^48^ hMOs from donor #2, with a looser, grape-like morphology, exhibited strong hypoxia and glycolysis signatures confirming the analysis from the combined dataset (Supplementary Fig. 3d–f).

Taken together, metastatic luminal A and B BC hMOs from three independent patients were stably cultured and expanded over extended periods and demonstrated proliferative phenotypes, with highly donor-specific differences in expansion rate and tumor marker expression.

### Smac mimetics induce cIAP1/2 degradation and donor-specific loss of cell viability in metastatic hMOs

**Fig. 3:**
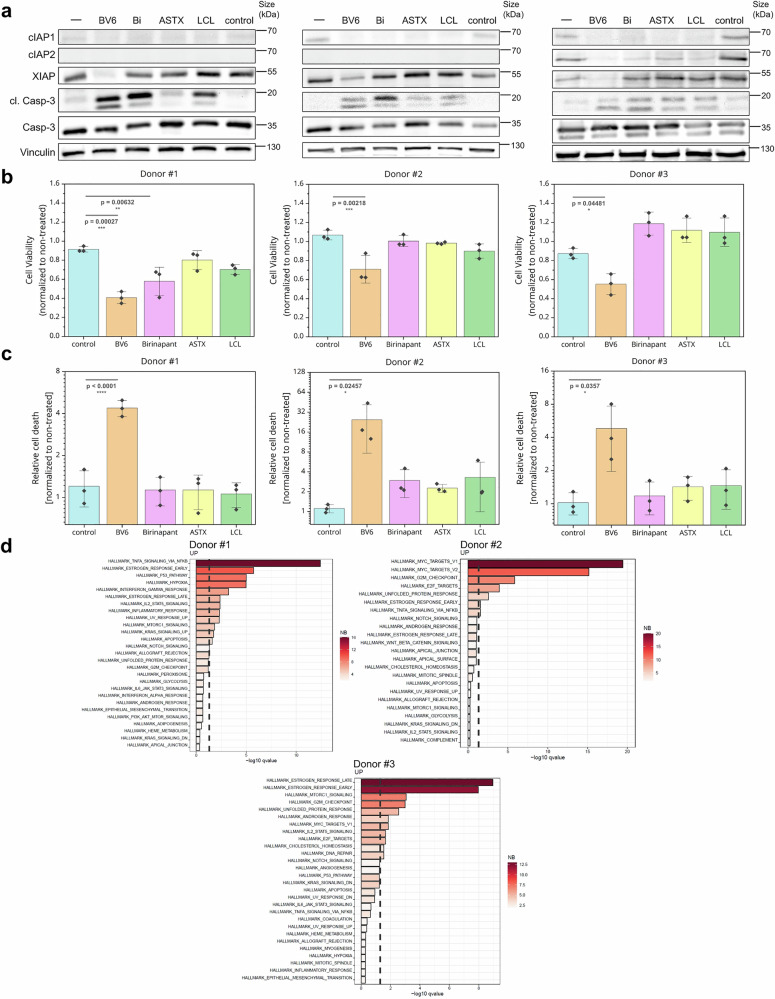
Smac mimetics induce cIAP1/2 degradation and donor-specific loss of cell viability in primary hMOs. hMOs were treated with 10 µM BV6, Birinapant, ASTX-660 and LCL-161, respectively or control-treated (0.4% DMSO). **a** Western blot analysis of cIAP1, cIAP2, XIAP, cleaved caspase-3, and full-length caspase-3 levels and vinculin as a loading control, was determined in non-treated and control-treated (0.4% DMSO) hMOs or after treatment with Smac mimetics for 24 h. **b** CellTiter-Glo® assay was performed and data were normalized to non-treated controls (*N* = 3). Error bars represent standard deviation. One-way ANOVA followed by Tukey’s test was used to calculate statistical significance. **** *p* < 0.0001; ***p ≤ 0.005; **p ≤ 0.01; *p ≤ 0.05; n.s. (not significant) *p* > 0.05. **c** Fluorescent live-dead assays were quantified, and data were normalized to non-treated control (*N* = 3). Error bars represent standard deviation. One-way ANOVA followed by Tukey’s test was used to calculate statistical significance. ****p < 0.0001; ***p ≤ 0.005; **p ≤ 0.01; *p ≤ 0.05; n.s. (not significant) *p* > 0.05. **d** Applying GSEA and the hallmark dataset, the most upregulated pathways upon Birinapant treatment per individual donor were identified. The x-axis represents the statistical significance of enrichment (–log₁₀ adjusted q-value), and the y-axis lists the top enriched gene sets. Bars are color-coded according to the number of differentially expressed genes (DEGs) contained within each gene set. The dashed vertical line indicates the threshold for statistical significance (–log_10_ adjusted *q*-value(0.05))

Having established a stable translational experimental pipeline to monitor patient-derived BC hMOs, we continued by investigating PCD sensitivities. Therefore, the basal, steady state expression of the apoptosis-related proteins caspase-3, cIAP1, and XIAP was quantified following Western blot analysis (Fig. 3a, Supplementary Fig. 4a). Overall, cIAP1 and XIAP showed low expression, whereas caspase-3 revealed highest expression with a statistically significant difference between hMOs from donor #1 and donor #3 (Supplementary Fig. 4a). Additionally, MLKL mRNA levels were comparable among all three donors (Supplementary Fig. 4b).

Following this, hMOs were treated with Smac mimetics, IAP antagonists that are applied to induce and sensitize towards PCD in solid tumor clinical trials.^17,18^ Stably growing hMOs were incubated with the Smac mimetics BV6, Birinapant (also referred to as TL32711), ASTX-660 (Tolinapant), and LCL-161 for 24 h, followed by Western Blot and cell viability measurement.

Western blot analysis of cIAP1, cIAP2, and XIAP expression in Smac mimetic-treated hMOs revealed compound-induced degradation of cIAP1 regardless of donor background compared to non-treated and control hMOs (Fig. 3a, Supplementary Fig. 4c). cIAP2 expression was only detected in hMOs from donor #3 and was strongly reduced by treatment with all tested Smac mimetics (Fig. 3a, Supplementary Fig. 4c). XIAP was expressed in all donors and potent loss of XIAP was observed by BV6 as well as the other Smac mimetics, although to a lesser extent (Fig. 3a, Supplementary Fig. 4c). Caspase-3 was detected in hMOs from all donors and Smac mimetics induced caspase-3 cleavage in a donor-independent manner, suggesting the induction of apoptosis (Fig. 3a, Supplementary Fig. 4c).

In all three donors, BV6 induced a significant reduction in cell viability compared to the DMSO vehicle control between 35% and 50%, whereas Birinapant, ASTX-660 and LCL-161 affected hMO viability in a highly donor-specific manner (Fig. 3b). In hMOs from donor #1 and #2, LCL-161 decreased the cell viability by approximately 15%, whereas this effect was absent in donor #3.

Although cell viability provides a good estimate of the cellular metabolic activity, it cannot differentiate between reduced metabolic activity due to, for example, lowered proliferation, or the actual induction of cell death. Therefore, time-lapse brightfield microscopy and image-based live-dead assays were performed using fluorescein diacetate (FdA) as a marker for living cells, propidium iodide (PI) as a marker for dead cells and Hoechst33342 (Hoechst) to monitor cells. Treatment of hMOs from all donors with Smac mimetics for 24 h induced prominent cell death, compared to non-treated or control conditions (Supplementary Fig. 5, 6). Furthermore, quantification of live-dead assays also revealed that BV6 triggered fourfold to 24-fold increases in dead cells compared to DMSO-treated control (Fig. 3c). The other Smac mimetics only induced smaller increases in dead cells compared to non-treated and controls (Fig. 3c).

To further investigate the cellular responses prior to actual cell death occurrence, the scCITEseq data were subjected to a donor-specific pseudo-bulk hallmark analysis. Upon treatment with Birinapant, donor #1 exhibited strong TNF-α signaling. Donor #2 showed prominent MYC signaling, whereas donor #3 mainly displayed estrogen response patterns (Fig. 3d). Pseudo-bulk analysis of the scCITEseq data with all donors combined showed high MYC signaling upon Birinapant treatment (Supplementary Fig. 4d), which confirmed that c-MYC overexpression might sensitize towards apoptosis, indicating a positive apoptotic feedback loop.^49^

### Smac mimetics induce PCD in patient-derived BC hMOs

BV6 and Birinapant demonstrated the strongest effects on hMO cell viability, though only Birinapant has been tested in clinical trials.^50,51^ Based on this, we further focused on Birinapant- and BV6-induced PCD in the BC organoids. hMOs were treated with the two Smac mimetics, the pan-caspase inhibitor Emricasan or DMSO as vehicle control, and subjected to time-lapse brightfield imaging.

**Fig. 4:**
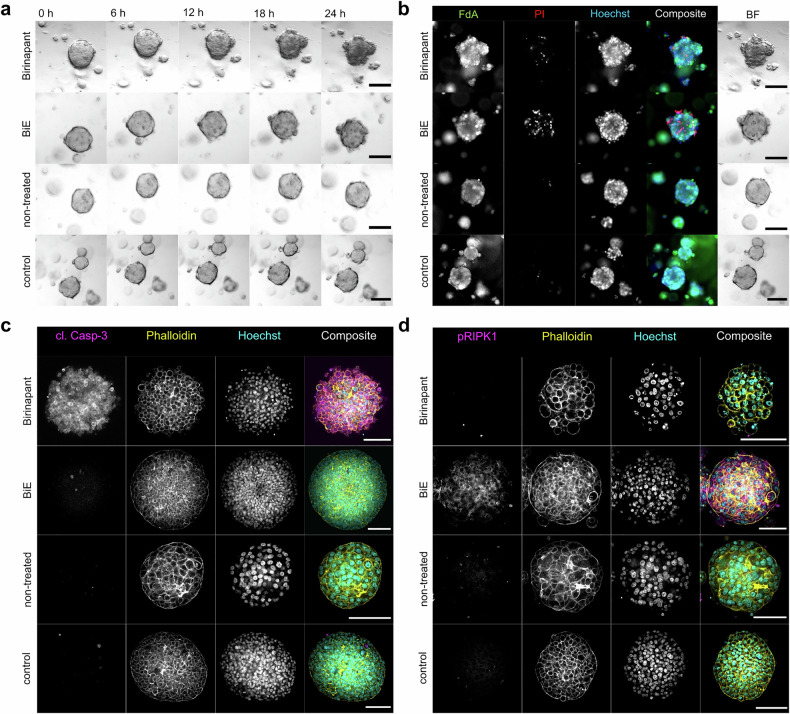
Smac mimetics induce PCD in primary hMOs. **a** Brightfield (BF) time-lapse images of non-treated, control (1.5% DMSO) and treated hMOs with 10 µM Birinapant or BiE (10 µM Birinapant and 10 µM Emricasan) were acquired using the Zeiss Z1 Axioimager widefield microscope every 30 min for 24 h and shown after 0, 6, 12, 18 and 24 h of imaging. **b** After 24 h live imaging, the same hMOs from (**a**) were stained using fluorescein diacetate (FdA, viable cells, green), propidium iodide (PI, dead cells, red) and Hoechst33342 (all nuclei, blue) and imaged again. **c**, **d** hMOs, treated for 24 h as in (**a**), were fixed and subjected to immunofluorescence staining against cleaved caspase-3 (**c**, cl. Casp-3, magenta) and S166-phosphorylated RIPK1 (**d**, pRIPK1, magenta) and counterstained using AF647™-Phalloidin (yellow) and Hoechst33342 (cyan). Organoids were cleared with CUBIC-2 and imaged using a Zeiss LSM780 confocal microscope. **a**, **d** Representative images of donor #1, scale bar: 100 μm

Treatment with Birinapant induced morphological alterations after 12 h with individual cells emerging from the compact organoids and the hMOs adopting a looser morphology, especially in donor #1 and #3 (Fig. 4a, Supplementary Fig. 7a, b). After 18 h, the formation of small vesicles resembling apoptotic bodies could be observed. Subsequent live-dead assays revealed increased cell death in Birinapant-treated hMOs compared to non-treated condition or controls (Fig. 4b, Supplementary Fig. 7c, d). Corresponding positive controls of DMSO-killed hMOs right before live-dead analysis are shown in Supplementary Fig. 8. As additional control for the induction of cell death, hMOs were treated with the anthracycline Doxorubicin for 72 h, following a live-dead analysis, showing induction of cell death with similar morphological alterations and increased numbers of PI-positive nuclei (Supplementary Fig. 9).

To model apoptosis resistance, caspases were inhibited by the pan-caspase inhibitor Emricasan. Birinapant- and Emricasan-cotreated hMOs (labeled as BiE) appeared more compact compared to the apoptotic hMOs with larger spheres emerging from the organoid, indicating cell swelling after 18 h (Fig. 4a, Supplementary Fig. 7a, b). Cotreated hMOs exhibited more prominent cell death, indicated by more PI-positive cells (Fig. 4b, Supplementary Fig. 7c-d).

To confirm the mode of PCD, hMOs were stained against cleaved caspase-3 as a marker for apoptosis and phosphorylated RIPK1 as an indicator for necroptosis. Only Birinapant-treated organoids showed caspase-3 cleavage, whereas RIPK1 phosphorylation was exclusively detected in Birinapant- and Emricasan-cotreated hMOs from all donors (Fig. 4c, d, Supplementary Fig. 10a, b). Cleaved caspase-3-positive organoids also exhibited a rounder cell shape based on the actin staining by phalloidin and condensed nuclei typical of apoptotic cells. Interestingly, hMOs from donor #3 showed some caspase-3 cleavage even under non-treated conditions which might correspond with lower expansion rates (Supplementary Fig. 10b). As observed in brightfield imaging, necroptotic hMOs showed a more compact morphology with individual cells undergoing swelling and expulsion from the organoid (Fig. 4d, Supplementary Fig. 10a, b).

Overall, BV6 induced similar effects to Birinapant but exhibited a slightly higher potency, indicated by the onset of cell death after 6 h (Supplementary Fig. 11a–c) and resulted in a stronger PI signal, indicating higher numbers of dead cells (Supplementary Fig. 11d–f). BV6 concentrations that induce cell death have been determined empirically (Supplementary Fig. 11g, h). Like in the Birinapant-treated hMOs, prominent caspase-3 cleavage was observed in the BV6-treated hMOs, whereas cotreatment with Emricasan led to RIPK1 phosphorylation (Supplementary Fig. 12).

These data suggest that cell death can be differentially induced and monitored in primary hMOs from multiple independent donors, and caspase inhibition can be applied to induce necroptosis and model apoptosis resistance in patient-derived organoids.

### Pharmacological modulation of LUBAC and necroptosis effectors rescues Birinapant- and Emricasan-induced PCD in hMOs

**Fig. 5:**
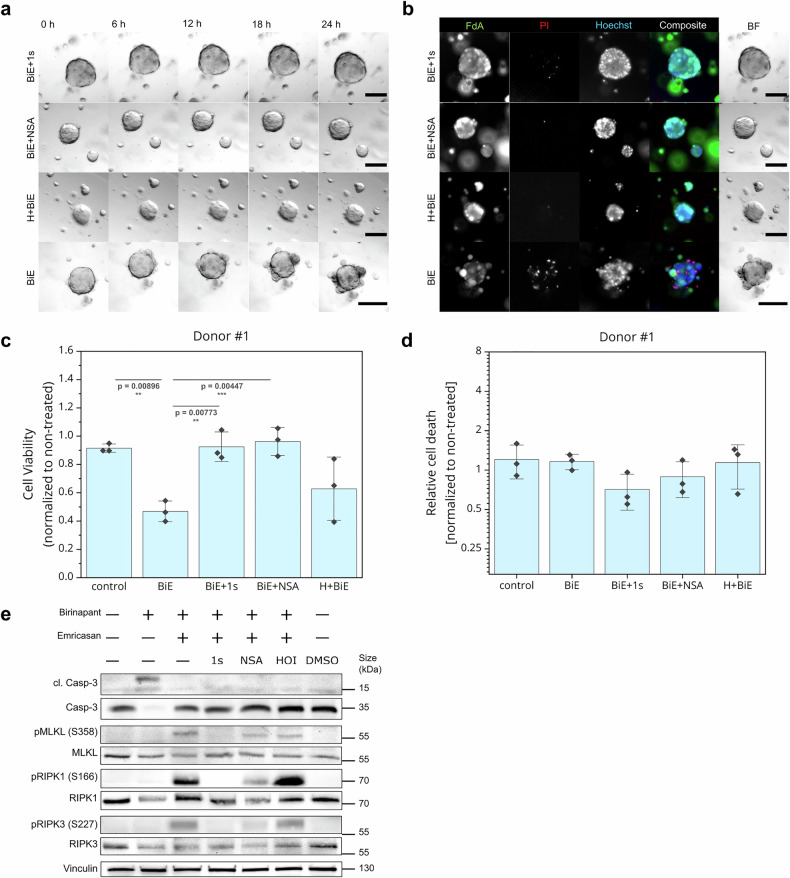
Pharmacological modulation of LUBAC and necroptosis effectors rescue Birinapant- and Emricasan-induced PCD in hMOs. **a** Brightfield time-lapse images of BiE-treated hMOs (10 µM Birinapant, 10 µM Emricasan) in combination with 1 s (30 µM necrostatin-1s), NSA (20 µM necrosulfonamide) or H (30 µM HOIPIN-8 with 30 min pre-treatment) were acquired in the Zeiss Z1 Axioimager widefield microscope with 30 min intervals for 24 h and shown after 0, 6, 12, 18 and 24 h of imaging. **b** After 24 h live imaging, the same hMOs from (**a**) were stained using fluorescein diacetate (FdA, viable cells, green), propidium iodide (PI, dead cells, red) and Hoechst33342 (all nuclei, blue) and imaged again. **a**, **b** Representative images of donor #1, scale bars: 100 µm. **c** CellTiter-Glo® viability assay was performed on hMOs from donor #1 treated as in (**a**). Values were normalized to non-treated controls. Error bars represent standard deviation. One-way ANOVA followed by Tukey’s test was used to calculate statistical significance. ****p < 0.0001; ***p ≤ 0.005; **p ≤ 0.01; *p ≤ 0.05; n.s. (not significant) *p* > 0.05. **d** Fluorescent live-dead assays, representative images in (**b**), were quantified and data were normalized against the non-treated condition (*N* = 3). One-way ANOVA followed by Tukey’s test was used to calculate statistical significance. ****p < 0.0001; ***p ≤ 0.005; **p ≤ 0.01; *p ≤ 0.05; n.s. (not significant) *p* > 0.05. **e** Western blot against phosphorylated and total RIPK1, RIPK3 and MLKL of hMOs from donor #1, treated as in (**a**). Vinculin served as a loading control. Representative blots of at least three independent experiments are shown

To investigate whether the experimental platform enabled pharmacological screening and whether necroptotic responses in hMOs could be rescued, the RIPK1 inhibitor necrostatin-1s, the MLKL inhibitor necrosulfonamide (NSA), and the LUBAC inhibitor HOIPIN-8 were evaluated on Birinapant- and Emricasan-treated hMOs. In addition to the necroptosis inhibitors, the LUBAC inhibitor HOIPIN-8 was chosen as it has been shown to attenuate PCD induction.^38^ Upon cotreatment with the inhibitors, no necroptosis-associated morphological changes were observed in the hMOs during time-lapse imaging (Fig. 5a, Supplementary Fig. 13a, b) and reduced numbers of dead cells were detected in the fluorescent live-dead assays (Fig. 5b, Supplementary Fig. 13c, d). Moreover, inhibition of RIPK1 and MLKL, and to a lesser extent also LUBAC, rescued hMO cell viability and cell death (Fig. 5c, d, Supplementary Fig. 14a–c), although hMOs from donor #3, which showed no reduction in cell viability and cell death when treated with Birinapant and Emricasan, were not affected upon incubation with the inhibitors (Supplementary Fig. 14b–d).

Western blot analysis revealed prominent induction of RIPK1, RIPK3, and MLKL phosphorylation in necroptotic hMOs from donors #1 and #2, which was absent in untreated, control- or Birinapant-treated hMOs (Fig. 5e, Supplementary Fig. 14e, 15a, b). As expected, inhibition of RIPK1 by Nec-1s prevented phosphorylation of RIPK1, RIPK3, and MLKL, whereas NSA did not affect phosphorylation of any, as it prevents necroptosis by binding MLKL at cysteine 86, inhibiting oligomerization and thus, interfering with MLKL membrane translocation and necroptotic pore formation.^52,53^ Interestingly, inhibition of LUBAC did not affect the necroptosis-associated phosphorylation patterns, confirming our previous observations that HOIPIN-8 inhibits necroptosis downstream of MLKL phosphorylation.^38^ Intriguingly, although necroptosis induction in hMOs from donor #3 did not reveal significant changes in cell viability or dead cells, phosphorylation of RIPK1 and RIPK3 could be detected (Supplementary Figs. 14b, d, f and 15c). In addition, necroptosis inhibitors did not affect cell viability or relative cell death. This is consistent with the findings that donor #3-derived BC organoids showed low MLKL protein levels, most likely only enabling very low levels of necroptotic cell death but still revealing necroptotic signaling upstream of MLKL.

Similar findings could be obtained upon treatment with BV6, Emricasan and necroptosis inhibitors (Fig. 4, Supplementary Figs. 7, 16 and 17).

Intriguingly, investigating the hallmark gene expression signatures of the HOIPIN-8-pretreated organoids from all donors combined revealed strong induction of the reactive oxygen species pathway (Supplementary Fig. 18a). Indeed, among the six most upregulated genes were genes involved in antioxidant processes, including *GCLM, GCLC* and *LUCAT1* (Supplementary Fig. 18b). *GCLM* and *GCLC* form the γ-glutamylcysteine synthetase which is known to be expressed in response to oxidative stress.^54^ Similarly, *LUCAT1* has been shown to be upregulated when cells where exposed to cigarette smoke-induced ROS.^55^ Cells that upregulated *LUCAT1* simultaneously upregulated genes involved in fatty acid metabolism, but downregulated genes involved in glycolysis. This agrees with our findings that revealed more prominent fatty acid metabolism compared to glycolysis under necroptotic conditions, whereas glycolysis was listed higher than fatty acid metabolism in control hMOs (Fig. 2d, Supplementary Fig. 18a). Comparing the different conditions with all donors combined, three clusters emerged that were mainly present in the HOIPIN-8 pretreated condition: clusters 4, 14 and 17 (Supplementary Fig. 18c). Analysis of the six most upregulated genes per cluster confirmed the induction of ROS signaling as *LUCAT1* and *HMOX1* were both included (Supplementary Fig. 18d–f). Moreover, in cluster 4, expression of *UCHL1* was demonstrated which is associated with resistance to chemotherapeutics (Supplementary Fig. 18d).

Similar findings were observed in the datasets from the individual donors (Supplementary Fig. 19a–c). Furthermore, other significantly upregulated pathways included MTORC1 signaling, UV response, xenobiotic metabolism, fatty acid metabolism and adipogenesis (Supplementary Fig. 19d–f). These findings highlight common effects of HOIPIN-8 on metastatic breast cancer, even with different characteristics and marker expressions.

Taken together, these data demonstrate strong induction of necroptosis with Smac mimetics and caspase inhibitors in primary BC hMOs, characterized by phosphorylation of RIPK1, RIPK3 and MLKL. Inhibition of RIPK1, RIPK3 or MLKL prevented necroptosis in primary hMOs, similarly as inhibition of LUBAC.

### Necroptosis induces the secretion of inflammatory mediators in metastatic hMOs

**Fig. 6:**
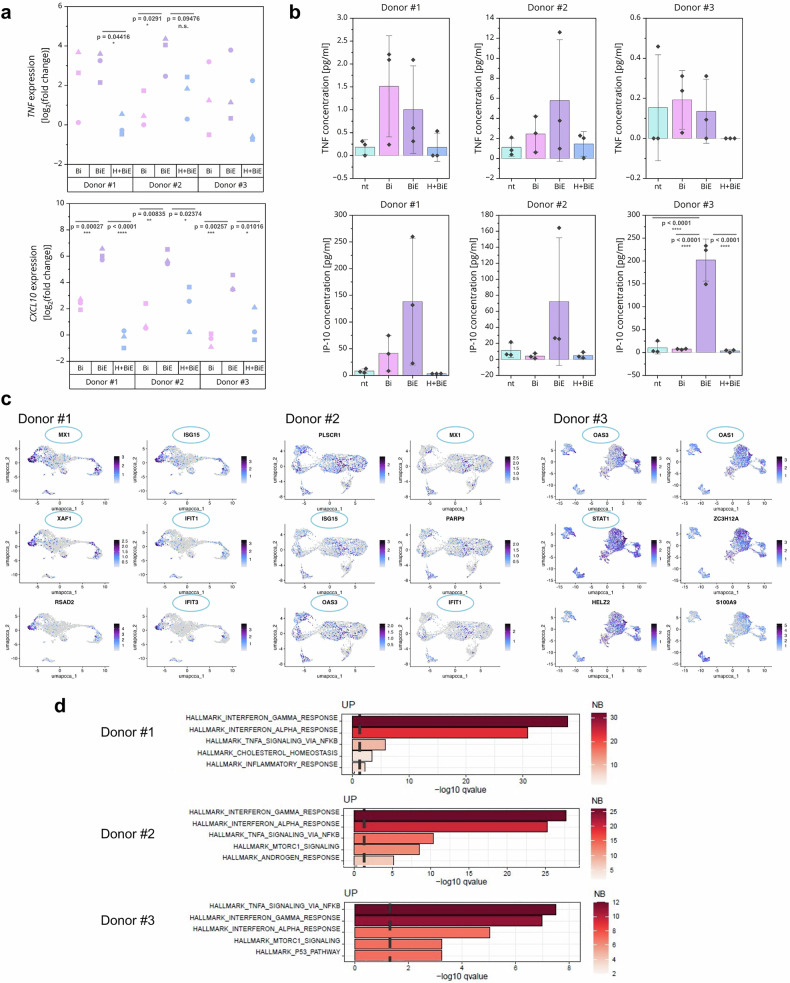
Necroptosis induces the secretion of inflammatory mediators in metastatic hMOs. **a** mRNA expression levels of *TNF* and *CXCL10* of HOIPIN-8 (30 µM) pre-treated hMOs upon treatment with 10 µM Birinapant (Bi) and 10 µM Emricasan (E) for 24 h. Gene expression was normalized to *RPII*, *18S-rRNA*, *TBP* and *RPL13* mRNA expression and is presented as log_2_(fold change). *N* = 3 independent experiments are shown. One-way ANOVA followed by Tukey’s test was used to calculate statistical significance. ****p < 0.0001; ***p ≤ 0.005; **p ≤ 0.01; *p ≤ 0.05; n.s. (not significant) *p* > 0.05. **b** TNF-α and IP-10 concentration in the supernatant from non-treated and treated hMOs measured by FACS-based CBA assay. Treatment was performed as in (**a**). *N* = 3 independent experiments are shown. Error bars represent the standard deviation. One-way ANOVA followed by Tukey’s test was used to calculate statistical significance. ****p < 0.0001; ***p ≤ 0.005; **p ≤ 0.01; *p ≤ 0.05; n.s. (not significant) *p* > 0.05. **c** UMAPs of the six most upregulated genes per donor upon 24 h treatment with 10 µM Birinapant and 10 µM Emricasan. Gene expression data from scCITEseq were analyzed and genes related to inflammation and interferon responses are circled in light blue. **d** Hallmark analysis of the scCITEseq dataset using GSEA per donor upon BiE-treatment for 24 h. The five most strongly induced signaling pathways are shown. The x-axis represents the statistical significance of enrichment (–log₁₀ adjusted q-value), and the y-axis lists the top enriched gene sets. Bars are color-coded according to the number of differentially expressed genes (DEGs) contained within each gene set. The dashed vertical line indicates the threshold for statistical significance (-log_10_ adjusted *q*-value(0.05))

Necroptosis is highly inflammatory, not only via the release of DAMPs upon membrane rupture, but also by transcription and secretion of inflammatory mediators, including cytokines and chemokines.^26^ Consistent with the induction of necroptosis, *TNF* and *CXCL10* mRNA expression was increased in Birinapant- and Emricasan-treated necroptotic hMOs from all three donors compared to Birinapant-treated apoptotic hMOs and non-treated conditions (Fig. 6a). Compared to the necroptotic condition, cotreatment with HOIPIN-8 decreased *TNF* and *CXCL10* mRNA expression to the expression level of non-treated hMOs in donor #1 and to the expression level of Birinapant-treated hMOs for donor #2 and #3 (Fig. 6a). Similarly, *CXCL1* and *ICAM1* mRNA expression showed the same increase upon induction of necroptosis and decreased upon necroptosis attenuation by HOIPIN-8, although with higher donor-to-donor variability compared to *TNF* and *CXCL10* (Supplementary Fig. 20a). Similar changes in gene expression were observed in hMOs treated with Smac mimetic BV6 (Supplementary Fig. 21).

Next, we investigated secretion of TNF-α and IP-10 during organoid necroptosis using cytometric bead array (CBA) FACS-based analysis (Fig. 6b). Overall, TNF-α concentrations in the supernatants were low but increased upon treatment with Birinapant or in combination with Emricasan. In all donors, pretreatment with HOIPIN-8 reduced TNF-α levels compared to Birinapant or BiE-treatment to approximately the same levels observed under non-treated conditions. IP-10, encoded by the gene *CXCL10*, showed overall higher concentrations, and treatment with Birinapant and Emricasan led to a strong increase in IP-10 secretion, which was reversed by pretreatment with HOIPIN-8. Similar responses were observed for secretion of pro-inflammatory IL-6 and IL-8 (Supplementary Fig. 20b). These results confirmed the attenuation of the necroptosis upon LUBAC inhibition, consistent with mRNA expression and previous findings.^38^

To further investigate the inflammatory responses of the BC hMOs upon necroptosis induction, the six most upregulated genes per donor were analyzed. Interestingly, high expression of interferon-stimulated genes (ISGs) could be detected in a donor-independent manner (Fig. 6c). hMOs from donors #1 and #2 expressed high levels of *MX1* and *IFIT1*, which are both involved in the antiviral response, similar to *OAS3*, which could be found in donors #2 and #3. Moreover, necroptotic hMOs from donor #3 highly expressed *STAT1*, whereas necroptotic organoids from donors #1 and #2 expressed high levels of *ISG15*. Pseudo-bulk hallmark analysis of the individual necroptotic donor organoids, and upon combining, confirmed strong induction of the interferon gamma (IFN-γ) and alpha (IFN-α) response as well as TNF-α signaling via NF-κB (Fig. 6d, Supplementary Fig. 20c–f).

In summary, our experiments revealed that induction of necroptosis in patient-derived hMOs increased ISG expression and interferon production that potentially contributes to necroptosis-associated inflammation, the TME, and anti-tumor immunity.

### Necroptotic hMOs induce NK cell responses

**Fig. 7:**
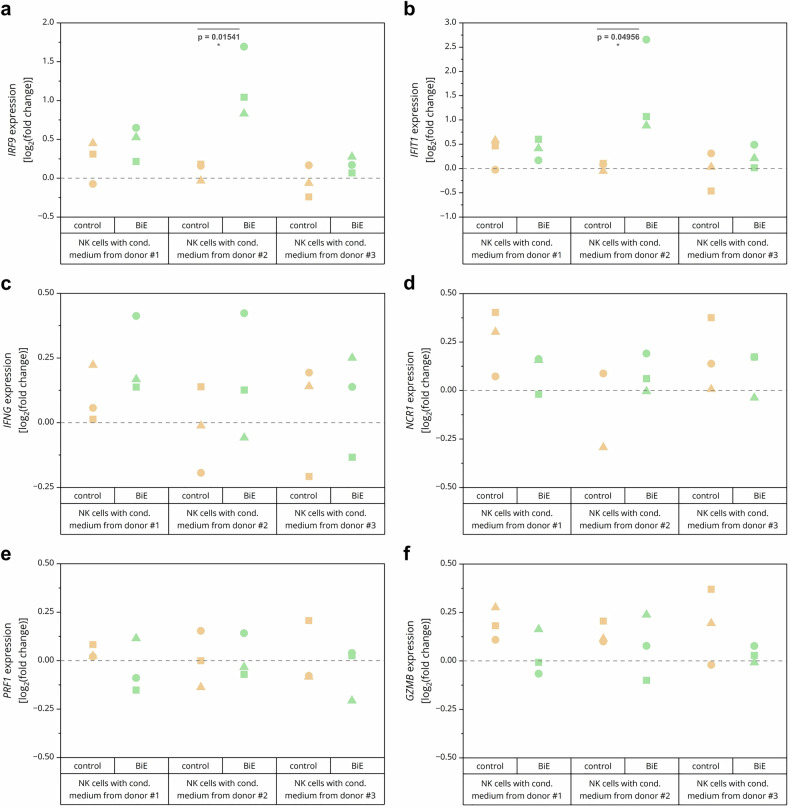
Necroptotic hMOs induce NK cell responses. *IRF9* (**a**), *IFIT1* (**b**), *IFNG* (**c**), *NCR1* (**d**), *PRF1* (**e**) and *GZMB* (**f**) mRNA expression levels upon treatment of NK cells with conditioned medium from hMOs for 4 h. Gene expression was normalized against non-treated conditions and reference genes *TBP*, *RPL13* and *RPII*. The data are presented as log_2_(fold change), and *N* = 3 independent experiments are shown. One-way ANOVA followed by Tukey’s test was used to calculate statistical significance. ****p < 0.0001; ***p ≤ 0.005; **p ≤ 0.01; *p ≤ 0.05; n.s. (not significant) *p* > 0.05

The transcriptional upregulation and secretion of necroptosis-induced inflammatory mediators might trigger immunogenic responses. To address this, NK-92 MI cells were incubated with conditioned medium from control and treated hMOs after wash-out of the necroptotic stimulus. NK cell mRNA was subsequently isolated, and genes were studied that are indicative of NK cell activation. NK cells treated with conditioned medium from necroptotic hMOs upregulated *IRF9* (coding for interferon regulatory factor 9), *IFIT1* (coding for interferon-induced protein with tetratricopeptide repeats 1) and to a lesser extent *IFNG* compared to NK cells treated with control conditioned medium (Fig. 7a–c), which was most prominent with conditioned medium from donor #2, highlighting the priming of the NK cells for activation. To further investigate NK cell cytotoxicity, RT-qPCR against *NCR1* (coding for natural cytotoxicity triggering receptor 1 also referred to as NKp46),^56^ *PRF1* (coding for perforin-1) and *GZMB* (coding for granzyme B),^57^ all markers for NK-mediated cytotoxicity, was performed. No major differences could be observed between control-treated and necroptotic hMO conditioned medium on the cytotoxic state of NK cells (Fig. 7d–f), indicating changes in NK cell signaling towards an activated state that has not reached functional cytotoxicity yet. These results indicate that induction of necroptosis targets apoptosis-resistant BC and may shape anti-tumor immunity, likely by priming immune cells for activation.

In summary, we describe a translational experimental pipeline to model and pharmacologically manipulate PCD responses, necroptosis-associated inflammatory signaling, and anti-tumor immunity in patient-derived metastatic BC organoids.

## Discussion

BC is the most common form of invasive cancer in women, with an increasing incidence. Despite advances in treatment options, relapse rates remain high due to primary and acquired chemoresistance.^2,3^ The study of necroptosis in human cancer settings is restricted to only a handful of necroptosis-proficient human tumor cell lines, which are still limited due to cell type-specific RIPK1, RIPK3, and MLKL expression. Thus far, primary and multicellular models that recapitulate complex cell-cell contacts, 3D interactions, and the physiological tissue-like cellular microenvironment to investigate the molecular mechanisms of necroptosis in human tissues are not available. Here, we describe a translational pipeline to model, mimic, and manipulate PCD sensitivities and associated inflammatory signaling in patient-derived, metastatic BC hMOs. This pipeline is available to further implement into drug screening processes and might be adapted to the specific study design, e.g., by including diverse methods such as proteomics or quantitative screening approaches.

3D cell and tissue cultures have emerged as a highly promising tool in cell biology and drug discovery.^4,5^ Multicellular human tumor organoids are driven by adult stem cells and represent a significant advancement in our ability to mimic the intricate functioning of tumors in vitro.^5,58^ In contrast to conventional 3D spheroids derived from tumor cell lines, primary tumor organoids offer a superior platform due to their unique composition, cultured from primary cells obtained directly from patients and maintained in a genetically stable and proliferative state for extended periods, often up to one year.^58^ Contrary to traditional 2D cultures, 3D cell cultures can be cultured in a chemically defined and GMP-compliant manner^59,60^ and provide more sophisticated and realistic representations of complex human physiology, tissue homeostasis, and cell-cell contacts.^6^ Consequently, this enhanced physiological relevance makes 3D cell cultures a valuable tool for investigating fundamental biological processes and understanding disease mechanisms with greater precision.

From an ethical standpoint, the utilization of 3D cell cultures offers a crucial opportunity to reduce the dependence on animal models in pre-clinical research.^58,59^ By employing human-derived 3D tumor organoids, the need for extensive animal testing can be bypassed, thus minimizing the ethical challenges and high cost associated with animal experimentation.^58^ This shift allows for a more cost-effective and efficient research approach. Furthermore, the establishment of more hMO lines from primary tumors as well as metastatic lesions from different BC subtypes would aid in understanding and pursuing more targeted approaches in clinically relevant human settings. Additional implementation and comparison between patients’ treatment responses, clinical data, and organoid models would further strengthen the conclusions.^5,61,62^

We established long-term, stable cultures of hMOs from metastatic lesions of luminal A (donors #2 and #3) and B (donor #1) BC patients. These hMOs differ morphologically from compact and round to grape-like and relatively fragmented, corresponding to the described morphological heterogeneity in BC organoids.^6,7,63^ In addition, these hMOs differ in the expression of E-cadherin and GATA-3. E-cadherin is a marker of low metastatic potential, and loss of E-cadherin has been associated with increased invasion,^64^ but reduced metastasis and reduced cell proliferation and survival.^65^ Of note, metastatic tumors often re-express E-cadherin at the metastatic site through mesenchymal-to-epithelial transition, thereby increasing proliferative capacity and survival.^66,67^ E-cadherin-negative hMOs from donor #2 displayed high proliferation rates and Ki67 expression. Since the effects of E-cadherin loss were attributed to increased reactive oxygen species (ROS) production and TGF-β signaling,^65^ including the antioxidant N-acetylcysteine and the TGF-β inhibitors A83-01 and Noggin, likely supported proliferation and cell survival of E-cadherin low-expressing hMOs.

GATA-3 is a transcription regulator required for normal mammary gland development and is associated with stemness and the luminal transcription program.^42^ GATA-3 expressing hMOs were more sensitive towards Birinapant- and Emricasan-induced necroptosis, indicating that GATA-3 expression might serve as a biomarker for PCD sensitivity in BC, but further research is required to confirm this finding.

To investigate the susceptibility of the hMOs towards PCD induction, we applied Smac mimetics. These substances degrade the IAP E3 ligases, thereby preventing pro-survival ubiquitination on cellular targets to induce cell death.^16,68^ Although suggested as novel treatment options for BC,^17,18^ in-depth investigations of Smac mimetics in patient-derived model systems were lacking. cIAPs and XIAP are reported to be overexpressed in BC^14^ and are associated with a worse prognosis.^2,69,70^ Treatment of hMOs from donor #3 led to a minor decrease in cell viability upon treatment with Birinapant, ASTX-660, and LCL-161. Overall, the bivalent Smac mimetics BV6 and Birinapant^71^ showed a stronger induction of PCD, whereas monovalent ASTX-660 and LCL-161 showed less potent effects, indicating a link between structure and potency. Comparing the two bivalent Smac mimetics, BV6 exerted stronger effects on the hMOs from all donors. This can be explained by the dissociation constants (Kd). BV6 has a Kd of 0.46 nM for cIAP1 and 0.029 nM for XIAP,^68^ whereas Birinapant exhibits Kds of <1 nM and 45 nM, respectively.^72^ Intriguingly, Birinapant, ASTX-660, and LCL-161 induced similar levels of caspase-3 cleavage compared to BV6, but with smaller effects on cell death and loss of cell viability. If this is caused by increased BV6-mediated XIAP degradation or by temporal or experimental-methodological factors remains unclear. All tested hMOs expressed TNF-α endogenously, and levels increased upon incubation with Smac mimetics. Under basal conditions, IAPs bind to NIK, and ubiquitination leads to NIK degradation.^73^ When IAPs are degraded by Smac mimetics, NIK is stabilized, leading to target gene expression involved in proliferation and cell death, including TNF-α.^74^ Hence, the increased expression levels of TNF-α drive feed-forward loops that support an inflammatory cellular microenvironment^26^ and the induction of apoptosis.^22^

Lytic forms of PCD, including necroptosis, are promising in overcoming apoptosis resistance observed in many tumors.^75^ Necroptosis is morphologically characterized by cell swelling, membrane rupture, and release of highly inflammatory cytokines, chemokines, and DAMPs.^25^ We modeled apoptosis resistance by applying pan-caspase inhibitor Emricasan, and the results demonstrate that apoptosis-resistant hMOs are susceptible to Smac mimetic-induced necroptosis. Interestingly, many BC cell lines do not express RIPK1, RIPK3, or MLKL and are non-sensitive towards necroptosis.^76^ Our findings demonstrated the expression and phosphorylation of RIPK1, RIPK3, and MLKL upon necroptosis progression in the hMOs, establishing primary, patient-derived organoids as a valuable model for BC necroptosis and RIPK1 phosphorylation as a biomarker. Our findings also reveal that necroptosis in hMOs induces the release of inflammatory mediators, such as IP-10, which has been shown to affect neighboring cells, the cellular microenvironment, and infiltration, differentiation, and function of immune cells.^75^ Moreover, we could show that necroptosis induced IFN-α and -γ signaling via scCITEseq analysis. These findings link necroptosis, IFN signaling, and release of cytokines for the first time in patient-derived BC organoids.

Furthermore, the role of linear ubiquitination and LUBAC in necroptosis was investigated. Although LUBAC is well characterized during early stages of TNF-α and Smac mimetic-induced cell death, the roles of linear ubiquitination in controlling necroptosis at the levels of MLKL remain largely unclear. LUBAC inhibition with HOIPIN-8 in the primary hMOs delayed necroptosis onset and lowered cytokine and chemokine expression and secretion. These results agree with a previous study investigating the influence of LUBAC inhibition on necroptosis in the human HT29 colon carcinoma cell line, as well as primary human pancreatic organoids.^38^ This might point towards tissue-, cell type- and species-specific modes of necroptosis regulation that further support the use of primary human tumor organoids as experimental models.

For many tumors, an immunosuppressive environment contributes to immune evasion failing to identify and target cancer cells.^77^ In BC, especially tumor-associated macrophages (TAMs) contribute up to 50% of the tumor cell mass and correlate with a poor prognosis.^78^ In our experimental platform, we showed that necroptosis induces cytokine expression and secretion that activates NK cells. Furthermore, the NK cells showed higher *IRF9* expression, a gene contributing to protection against apoptosis.^79^ These results demonstrate that necroptosis activates immune cells from solid BC tumor tissue that might promote anti-tumor immunity, tumor cell killing and improve immunotherapy.

Overall, our findings reveal primary patient-derived BC hMOs as a suitable model to study PCD sensitivities and inflammatory signaling and provide an attractive experimental platform for personalized and precision translational approaches, immunotherapy, and drug screening. By inducing necroptosis, we identified ISGs and interferon signaling as one of the drivers of the inflammatory response. Enhancement of anti-tumor immunity might be studied in advanced organoid-immune cell co-culture systems and might improve patient treatment, especially in apoptosis-resistant BC.

## Materials and methods

### Human mammary organoid source and ethics

Human mammary organoids (hMOs) were obtained from HUB Organoid Technology (Utrecht, Netherlands) (donor #1 refers to organoid line HUB-C2-120, #2 to HUB-C2-152, and #3 to HUB-C2-123, as published^6,7^). The use of the patient-derived hMOs has been approved by the medical ethical committee of the UMC Utrecht (METC UMCU; 12-427/C) under biobanking protocol HUB-Cancer TcBio#20-425. All patients signed informed consent forms, and the experiments were performed in accordance with the relevant guidelines and regulations. More information about the hMO lines can be found in Sachs et al. ^6^ or Dekkers *et al*. ^7^ or might be directly requested from Merck/HUB Organoids.

### Chemicals and reagents

BV6 was kindly provided by Genentech Inc. (San Francisco, CA, USA), Birinapant, ASTX-660, LCL-161, and the pan-caspase inhibitor Emricasan were purchased from Selleckchem (Houston, TX, USA) Nec-1s and NSA from Merck KGaA (Darmstadt, Germany), HOIPIN-8 from Axon Medchem LLC (Reston, VA, USA) and DOXO-cell® (doxorubicin hydrochloride) from STADA Arzneimittel (Bad Vilbel, Germany). All other chemicals were purchased from Carl Roth (Karlsruhe, Germany) or Merck KGaA (Darmstadt, Germany) unless stated otherwise. All media and supplements were purchased from ThermoFisher Scientific (Waltham, MA, USA) unless stated otherwise.

### Maintenance of hMOs

Patient-derived hMOs were kept in an incubator at 37°C in a humidified atmosphere with 5% CO_2_ and were cultured, in adapted form, as described previously.^6,7^ hMOs from donors #1 and #2 were grown in modified Type I organoid medium and hMOs from donor #3 were maintained in modified Type II organoid medium (Supplementary Table 1). Depending on proliferation rate and growth, organoids were passaged every seven to 21 days in a ratio of 1:2 – 1:8.

For passaging, hMOs were collected, washed with wash medium (Dulbecco’s Modified Eagles Medium with GlutaMAX supplement (DMEM,) supplemented with 0.1% Albumin Fraction V (BSA) and 100 U/ml Penicillin-Streptomycin (P/S)) using centrifugation and the wash was repeated using basal medium (Advanced Dulbecco’s Modified Eagles Medium with Nutrient Mixture F-12 supplemented with 1 M HEPES, 1x GlutaMAX and 100 U/ml P/S). The organoid pellet was resuspended in basal medium and TrypLE™ Express Enzyme and mechanically sheared into small fragments by pipetting. Fragmented organoids were pelleted in basal medium and subsequently resuspended in 20% organoid medium with 80% Cultrex Reduced Growth Factor Basement Membrane Extract, Type 2 (BME, R&D Systems), depending on the passaging ratio. BME droplets were placed in pre-warmed CELLSTAR® suspension culture plates (35 µl per well in a 24-well plate or 5 µl per well in a 96-well plate; Greiner Bio-One GmbH, Frickenhausen, Germany) and after solidification, the droplets were overlaid with organoid medium (500 µl for a 24-well plate, 100 µl for a 96-well plate).

### Treatment of hMOs

hMOs were expanded for three to seven days, followed by 30 min pre-treatment with HOIPIN-8 as indicated. Pursuant to this, hMOs were incubated for 24 h with the Smac mimetics BV6, Birinapant, ASTX-660 or LCL-161, the RIPK1 inhibitor necrostatin-1s (Nec-1s), the MLKL inhibitor necrosulfonamide (NSA) or the pan-caspase inhibitor Emricasan (E) in the indicated concentrations.

For treatment with DOXO-cell® (doxorubicin), hMOs were expanded as described above and incubated with the indicated concentrations for 72 h.

### Time-lapse imaging and live-dead assays

For time-lapse imaging, the hMOs were transferred to the Zeiss Z1 Axioimager widefield microscope (37 °C, 5% CO_2_) and images were acquired every 30 min for a total period of 24 h with a 2 × 2 tiling and a Z-stack of 11 slices with 60 µm spacing.

For live-dead assays, hMOs were stained using 10 µg/ml propidium iodide (PI, Millipore Sigma), 0.5 µg/ml fluorescein diacetate (FdA, Millipore Sigma), and 200 µg/ml Hoechst33342 (ThermoFisher Scientific). The hMOs were washed with phosphate-buffered saline (PBS), followed by imaging, applying the same settings as above. Images were exported using the ZEN 2.6 lite software (Carl Zeiss Microscopy GmbH) and subsequently processed using the FiJi/ImageJ software.

For quantification of live-dead assays, a FiJi/ImageJ macro has been used. In brief, the 11 z slices per tile were processed to a maximum projection, and the background, which had been manually calculated beforehand, was subtracted from each image. Then, the average intensity was measured per tile and averaged. The data were normalized on the corresponding technical replicates (*N* = 3) of the non-treated conditions, and in total, three biological replicates have been processed per donor. The data are presented in bar plots overlaid with the individual data points using Origin 2023b Pro, and the error bars represent the standard deviation.

### Viability measurements

CellTiter-Glo® Luminescent Cell Viability (CTG, Promega) assays were performed after 24 h treatment following manufacturer’s instructions. The data were normalized against non-treated conditions and presented in bar plots with the standard deviation of three biological replicates using Origin 2023b Pro.

### hMO lysis and Western blotting

For Western Blot, hMOs were expanded for up to ten days to reach high confluency. After treatment, multiple wells per condition were pooled, and organoids were harvested. To remove the BME, organoid pellets were washed with wash and basal medium, followed by PBS, and stored at –20 °C until further processing. hMO pellets were resuspended in RIPA lysis buffer (ThermoFisher Scientific) supplemented with PhosSTOP™ (Roche, Basel, Switzerland) and Protease Inhibitor Cocktail (Sigma) for 20 min on ice. The suspension was centrifuged to remove cell debris. Protein concentrations were determined in cleared lysates using the BCA Protein Assay kit (ThermoFisher Scientific) following the manufacturer’s protocol and using an infinite M200 plate reader (Tecan, Männedorf, Switzerland).

hMO lysates were diluted in water and denatured in 4x Laemmli buffer (ThermoFisher Scientific) with β-mercaptoethanol (Bio-Rad Laboratories, Hercules, CA, USA) to a final concentration of 1 mg/ml by incubating at 85 °C at 300 rpm for 10 min, followed by SDS-PAGE. The antibodies are listed in Supplementary Table 2. The following horseradish-peroxidase-coupled secondary antibodies were used for detection (Supplementary Table 3) using Pierce™ ECL Western Blotting-Substrate (ThermoFisher Scientific). Representative blots of at least two independent experiments are shown. If the samples of one experiment are detected on multiple Western blotting membranes, only one representative loading control is shown for clarity. Original Western blot images were inverted for clarity and processed using the FiJi/ImageJ software.

For quantification of the Western blots, the images were loaded into Licor iStudio (LI-COR GmbH, Lincoln, NE, USA), and rectangles were drawn around the bands. For each antibody, the same-sized rectangle was applied per blot. The calculated signals for the bands of interest were normalized against the loading control, and data are presented as bar plots overlaid with the individual data points using Origin 2023b Pro. The error bars represent the standard deviation.

### RNA isolation, cDNA synthesis and quantitative real-time PCR

hMOs were expanded for up to seven days to high confluency. After the indicated treatments, the hMO-containing BME droplets were resuspended in 500 µl TRIzol reagent (ThermoFisher Scientific) and collected at –20 °C. RNA extraction was conducted following the manufacturer’s instructions.

Total RNA concentrations were measured using NanoPhotometer® (Implen, Westlake Village, CA, USA), and 1.5 to 3 µg RNA were used for cDNA synthesis using the Maxima First Strand cDNA Synthesis Kit with dsDNAse (ThermoFisher Scientific) according to the manufacturer’s instructions.

For RT-qPCR, PowerTrack™ SYBR Green MasterMix (ThermoFisher Scientific) was used with a concentration of 5 ng of cDNA and 400 nM per primer per 10 µl reaction volume, using the CFX96 C1000 Touch qPCR system (BioRad). Data were normalized against *TBP, RPL13, 18S-rRNA*, and *RPII* expression, and relative gene expression levels were calculated using the ΔΔCt-method. The primers used in this study were designed using PrimerBlast^80^ and are listed below. Primers were obtained from Eurofins (Hamburg, Germany) and Biomers (Ulm, Germany), and the sequences can be found in Supplementary Table 4.

### Immunofluorescence staining and confocal microscopy

hMOs were grown to medium confluency. After treatment, hMOs were fixed using 4% paraformaldehyde (PFA, Electron Microscopy Sciences) on ice and washed three times using ice-cold PBS. For immunostaining, organoids were permeabilized with 0.3% Triton X-100 in PBS for 40 min at RT and subsequently washed with 100 mM glycine in PBS, followed by PBS supplemented with 0.1% Triton X-100 and 2% P/S (PBS-T). Organoids were blocked in 0.2% Triton X-100, 0.1% Tween-20, 0.1% BSA, 2% P/S, and 10% donkey serum (Sigma) in PBS. Primary antibody solutions in blocking solution were incubated overnight at 37 °C and 300 rpm. The following day, organoids were washed with PBS-T and incubated with the secondary antibodies in blocking solution supplemented with 2 µg/ml Hoechst33342 (H1399, ThermoFisher Scientific) and 132 nM AlexaFluor™488 Phalloidin (A12379, ThermoFisher Scientific). The antibodies used in this study are listed in Supplementary Tables 2 and 3. Prior to imaging, organoids were washed again and transferred onto a thin layer of CUBIC-2 (15% w/w MilliQ H2O, 50% w/w Sucrose, 25% w/w Urea, 10% w/v Triethanolamine) on a microscope slide (Epredia, Portsmouth, NH, USA). A cover glass was placed on top, and the organoids were imaged using a Zeiss LSM780 confocal microscope with a Plan-Apochromat 20x/0.8 M27 objective. Image processing was performed using Zeiss ZEN 3.8 and FiJi/ImageJ software.

### Cytometric Bead Array (CBA) assays

hMOs were seeded in 10 µl droplets in a 96-well plate and expanded to high confluency. Organoids were treated in 70 µl medium for 24 h, after which supernatants were collected and stored at -80°C. Assays were performed with the BD® Cytometric Bead Array (CBA) assay, according to the manufacturer’s instructions. The cytokine- and chemokine levels were determined using FACSymphony™ A5 SE (BD). Analysis was performed using FlowJo Software (BD) and visualized using Origin.

### Single-cell Cellular Indexing of Transcriptomes and Epitopes by Sequencing (scCITEseq).^81^

scCITEseq was performed as described previously.^81^ Briefly, control and treated organoids were harvested and disintegrated into single cells with undiluted TrypLE and mechanical shearing. Single cell suspensions were passed through a single cell strainer (Falcon® 5 mL Round Bottom Polystyrene Test Tube, with Cell Strainer Snap Cap) and stained using the Invitrogen™ LIVE/DEAD™ Fixable Red Dead Cell Stain Kit following the manufacturer’s instructions. For multiplexing, sample tags were added to the cells in BD Pharmingen™ Stain Buffer (BD) with BSA. After staining, 100,000 viable cells per condition per donor were collected using fluorescence-activated cell sorting, and all conditions per donor were merged. From this point on, all cells were kept on ice.

#### Single-cell capture and barcoding

The sorted cells were washed and resuspended in Stain Buffer prior to adding the premixed antibody-derived tags (ADTs, BD, Supplementary Table 5). After incubation for 40 minutes on ice, cells were washed with BD Stain Buffer and then resuspended in BD sample buffer (BD).

As control, a portion of the cells were stained with 2 mM Calcein AM (BD) and 2 mM Draq7 (BD), and cell viability was analyzed in a disposable hemocytometer (Incyto) using the Rhapsody scanner (BD). Following this, the cell suspension was loaded on the Rhapsody microwell cartridge (BD) to capture 40,000 cells according to the manufacturer’s instructions. Oligonucleotide-labeled beads were added, incubated and washed prior to cell lysis by 5 mM DTT. After bead removal, the cDNA was synthesized using the corresponding cDNA kit (BD).

#### Library preparation and sequencing

The DNA libraries were produced according to the instructions of the BD Rhapsody whole transcriptome analysis (WTA), ADTs, and Sample Tag amplification kit (BD). In a last amplification step, indexes for Illumina sequencing were included in the final libraries before assessing their concentration and size with a Qubit 4 Fluorometer using a High Sensitivity dsDNA kit (ThermoFisher Scientific) and an Agilent 4150 TapeStation using a High Sensitivity D1000 ScreenTape (Agilent, Santa Clara, CA, USA). Finally, library pools with a final concentration of 1 nM were combined and loaded on a NextSeq2000 sequencer (Illumina) with standard settings sequencing for 75 bp paired-end, together with 20% PhiX DNA (Illumina).

#### Read alignment and quantification

Following Illumina sequencing, the FASTQ files and reference sequences for mRNAs and ADTs were uploaded to the SevenBridges platform (Seven Bridges Genomics, https://www.sevenbridges.com). The data was processed using the BD Rhapsody analysis pipeline with default settings.

### Analysis of scCITEseq data

#### Pre-processing of the raw data

scCITEseq raw counts were processed using Seurat 5.1.0.^82^ Gene expression data were used to create a Seurat object, while ADTs were stored as separate assays within the same object. Data from different donors were split into individual layers to facilitate integration at later stages.

Quality control (QC) filtering was applied as follows: cells with nFeature RNA between 200 and 5000, nCount RNA below 20,000, mitochondrial content below 15%, ribosomal content below 15%, and cell complexity (log10(nFeature RNA)/ log10(nCount RNA)) above 0.8 were retained. Mitochondrial, ribosomal, and housekeeping genes were excluded from downstream analyses. In the final step, RNA-seq data were log-normalized using a natural logarithm transformation, and ADT data were normalized using centered log-ratio (CLR) normalization.

Data from individual donors were analyzed either independently or jointly. For integration, the IntegrateLayers function was applied using the CCAIntegration method with PCA as the dimensionality reduction approach. A nearest neighbor graph was constructed using the top 15 principal components (top 10 for individual donor analysis), and Louvain clustering was performed with a resolution set to 1 (0.8 for individual donor analysis). To visualize the remaining cells in two dimensions, Uniform Manifold Approximation and Projection (UMAP) was employed.

#### Markers

Marker detection was performed using the FindAllMarkers function with the Wilcoxon test. Minimum thresholds for expression percentage and log fold change were both set to 0.25. Markers were identified for both experimental conditions and Louvain clusters.

#### Fisher test

Pseudo-bulk gene set enrichment analysis was conducted using Fisher’s exact test on the top 100 markers from the clustering results.

#### Decoupler

Single-cell resolution gene set enrichment analysis was performed using the ULM method in the DecoupleR package. Gene set information was sourced from MSigDB^45,83,84^ and literature-derived PAM50 gene sets.^85^ For marker detection and gene set enrichment analyses, multiple testing correction was applied using the Benjamini-Hochberg (BH). Hallmark analysis following gene set enrichment analysis was performed and is presented as bar plots, whereas the x-axis represents the statistical significance of enrichment (–log₁₀ adjusted q-value), and the y-axis lists the top enriched gene sets. Bars are color-coded according to the number of differentially expressed genes (DEGs) contained within each gene set. The dashed vertical line indicates the threshold for statistical significance (-log_10_ adjusted q-value(0.05)).

### NK cell activation assay with hMO conditioned medium

hMOs were seeded in 10 µl drops in a 96-well plate and grown to high confluency. Organoids were treated in 70 µl media, and supernatants were discarded. 70 µl organoid media was added to each well and incubated for 24 h to generate conditioned media. The supernatants were collected and stored at –80 °C until further usage.

800,000 NK-92 MI cells were seeded in 900 µl NK medium (Supplementary Table 6) and treated with 100 µl hMO conditioned media for 4 h. Afterwards, total RNA was isolated with the RNeasy mini kit (QIAGEN, Hilden, Germany) following the manufacturer’s instructions. Extracted RNA was stored at –80 °C, and cDNA was synthesized using the Maxima First Strand cDNA Synthesis Kit with dsDNAse (ThermoFisher Scientific) according to the manufacturer’s instructions, followed by RT-qPCR as described above. The primers can be found in Supplementary Table 4.

### Statistical analysis

Statistical significance was determined using one-way ANOVA followed by Tukey’s test. p values < 0.05 are considered significant and depicted as follows: ****p < 0.0001; ***p ≤ 0.005; **p ≤ 0.01; *p ≤ 0.05; n.s. (not significant) *p* > 0.05.

## Supplementary information

Supplementary Material

Supplementary Data

## Data Availability

The data generated in this study is presented in the manuscript and supplementary materials. Primary data are uploaded to Zenodo (10.5281/zenodo.18504530), and scCITEseq data is deposited at GEO (GSE320583: GSM9546004, GSM9546005, GSM9546006). Data will be made publicly available upon publication of the manuscript.
