# Supplementary material for: Necroptosis triggers inflammatory interferon signatures in patient-derived metastatic breast cancer organoids
Source: Signal Transduct Target Ther. 2026 Jun 1;11:204. doi: 10.1038/s41392-026-02755-9 (PMC13226679; doi:10.1038/s41392-026-02755-9)
Supplement: Supplementary file 1 — Supplementary Material [file 41392_2026_2755_MOESM1_ESM.docx]

Supplementary Materials for

**Necroptosis triggers inflammatory interferon signatures in patient-derived metastatic breast cancer organoids**

Kaja Nicole Wächtershäuser, Jana V. Schneider, Alec Gessner, Geoffroy Andrieux, Ivan-Maximiliano Kur, Nadine Duschek, Andreas Weigert, Melanie Boerries, Michael A. Rieger, Ernst H.K. Stelzer, Francesco Pampaloni, Sjoerd J.L. van Wijk

Correspondence to: [s.wijk@kinderkrebsstiftung-frankfurt.de](mailto:s.wijk@kinderkrebsstiftung-frankfurt.de), [fpampalo@bio.uni-frankfurt.de](mailto:fpampalo@bio.uni-frankfurt.de).

**This PDF file includes:**

Supplementary Figures 1 to 21

Supplementary Tables 1 to 6

**Other Supplementary Materials for this manuscript include the following:**

Supplementary Data 1 to 7

**Supplementary Figure 1**

**
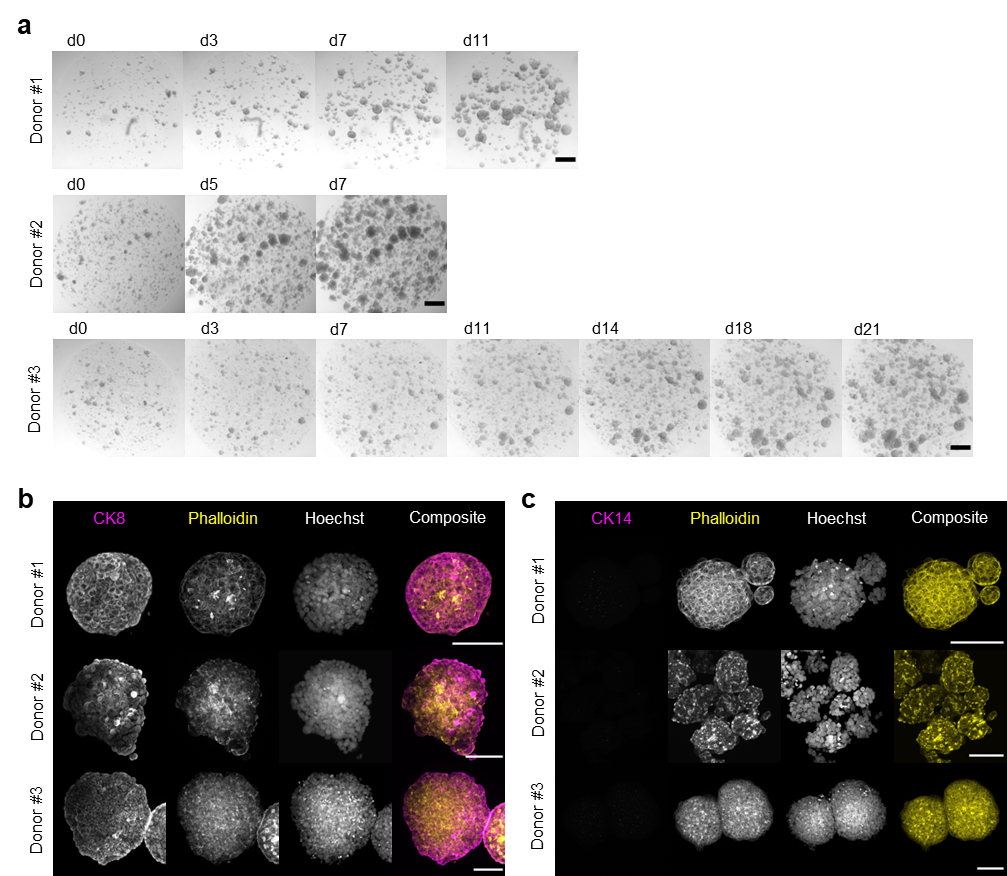
Supplementary Fig. 1 related to Fig. 1:** **hMOs show different expansion rates and express cytokeratin-8, but not cytokeratin-14.** (**a**) hMOs were imaged in brightfield for the indicated days until they reached confluency using the Zeiss SteREO Discovery.V8 microscope. Scale bars: 500 µm. (**b-c**) Immunofluorescent staining against cytokeratin-8 (**b**, CK8, magenta) or cytokeratin-14 (**c**, CK14, magenta) in hMOs from all three donors. Fixed organoids were counterstained using AF647™-Phalloidin (**b**, yellow) or AF488™-Phalloidin (**c**, yellow) and Hoechst33342, CUBIC-2 cleared and imaged using a Zeiss LSM780 confocal microscope. Maximum Z projection was created using FiJi. Scale bars: 100 µm.

**Supplementary Figure 2**

**
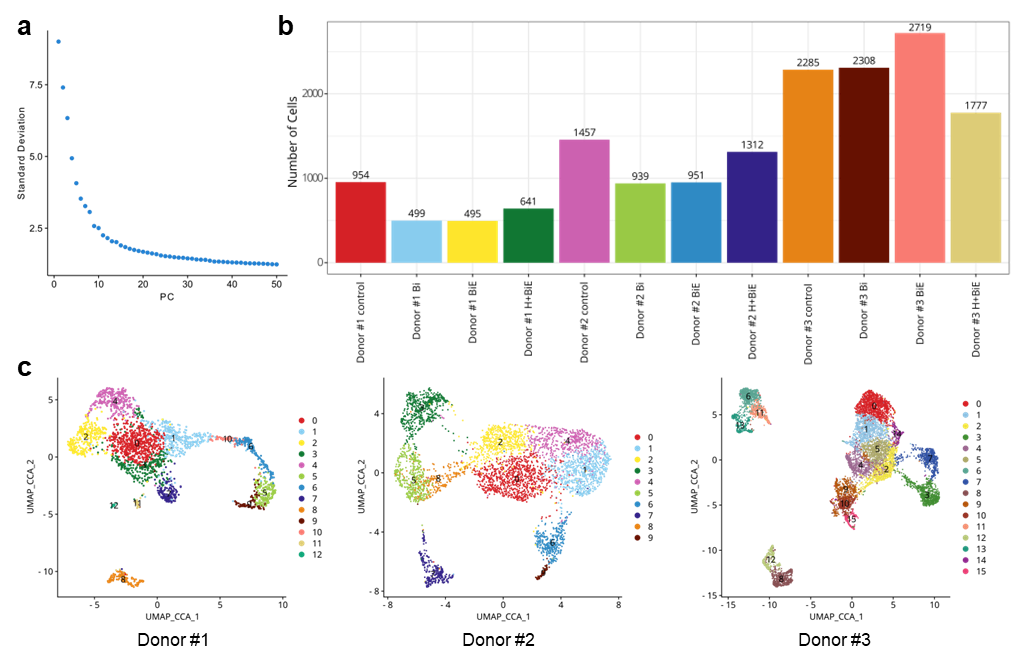
Supplementary Fig. 2 related to Fig. 1: Analysis of scCITEseq reveals 22 clusters.** (**a**) Elbow plot of the principal component analysis of the scCITEseq from all donors and conditions merged. Top 20 principal components were used for downstream analysis. (**b**) Cell numbers per condition from the three different donors from a total of 16,337 cells analyzed. hMOs were treated with 1.5 % DMSO as vehicle control, 10 µM Birinapant, 10 µM Emricasan (BiE) and pre-treated with 30 µM HOIPIN-8 (H+BiE) for 24 h. (**c**) UMAP clustering of individual donors.

**Supplementary Figure 3**

**
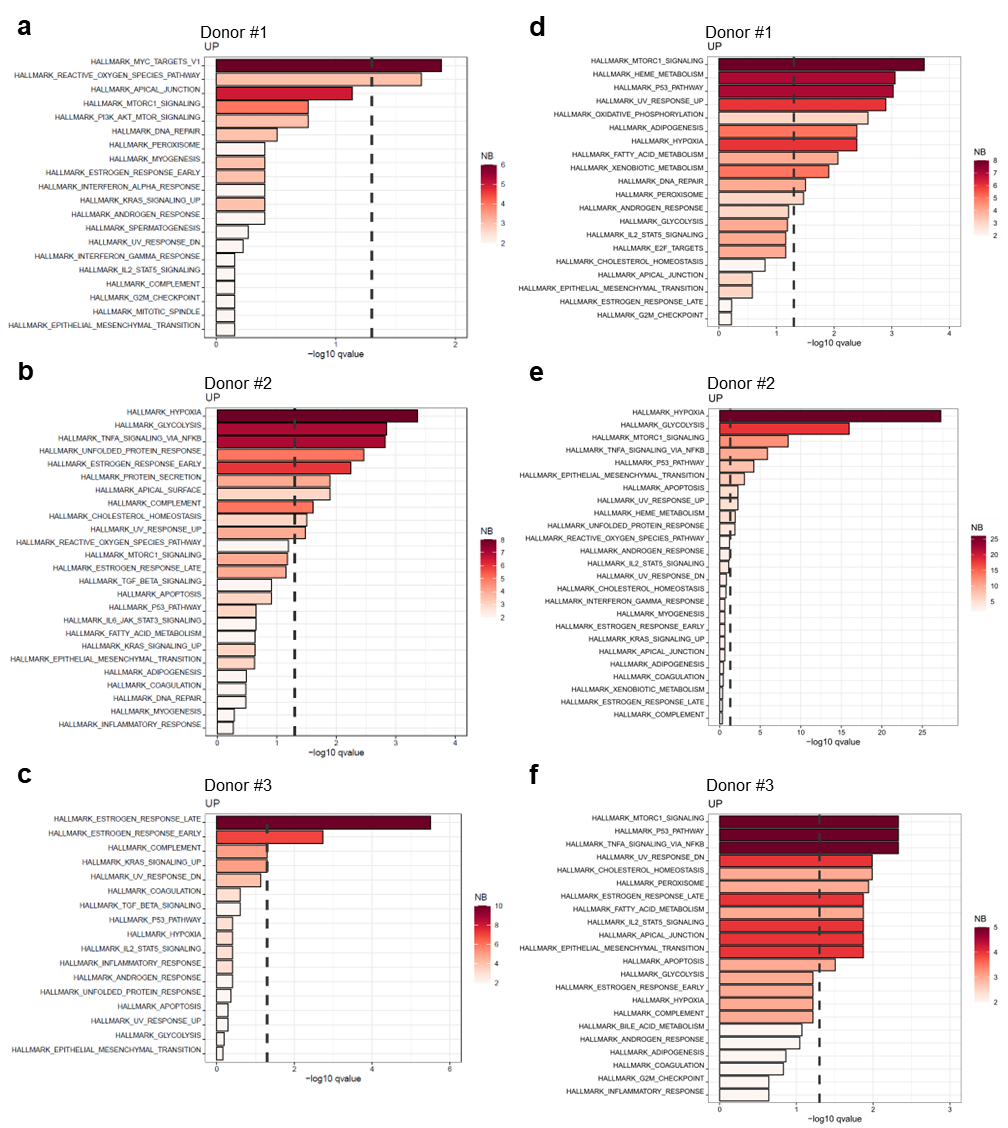
Supplementary Fig. 3 related to Fig. 2:** **Donor-specific hallmarks in control-treated hMOs.** (**a-c**) GSEA and hallmark analysis were applied and compared to all conditions. Control-treated hMOs (1.5 % DMSO) from donor #1 (**a**), #2 (**b**) and #3 (**c**) are shown. (**d-f**) The scCITEseq dataset was split per donor and the analysis was repeated individually on control-treated hMOs from donor #1 (**d**), #2 (**e**) and #3 (**f**). (**a-f**) The x-axis represents the statistical significance of enrichment (–log₁₀ adjusted q-value), and the y-axis lists the top enriched gene sets. Bars are color-coded according to the number of differentially expressed genes (DEGs) contained within each gene set. The dashed vertical line indicates the threshold for statistical significance (-log_10_ adjusted q-value(0.05)).

**
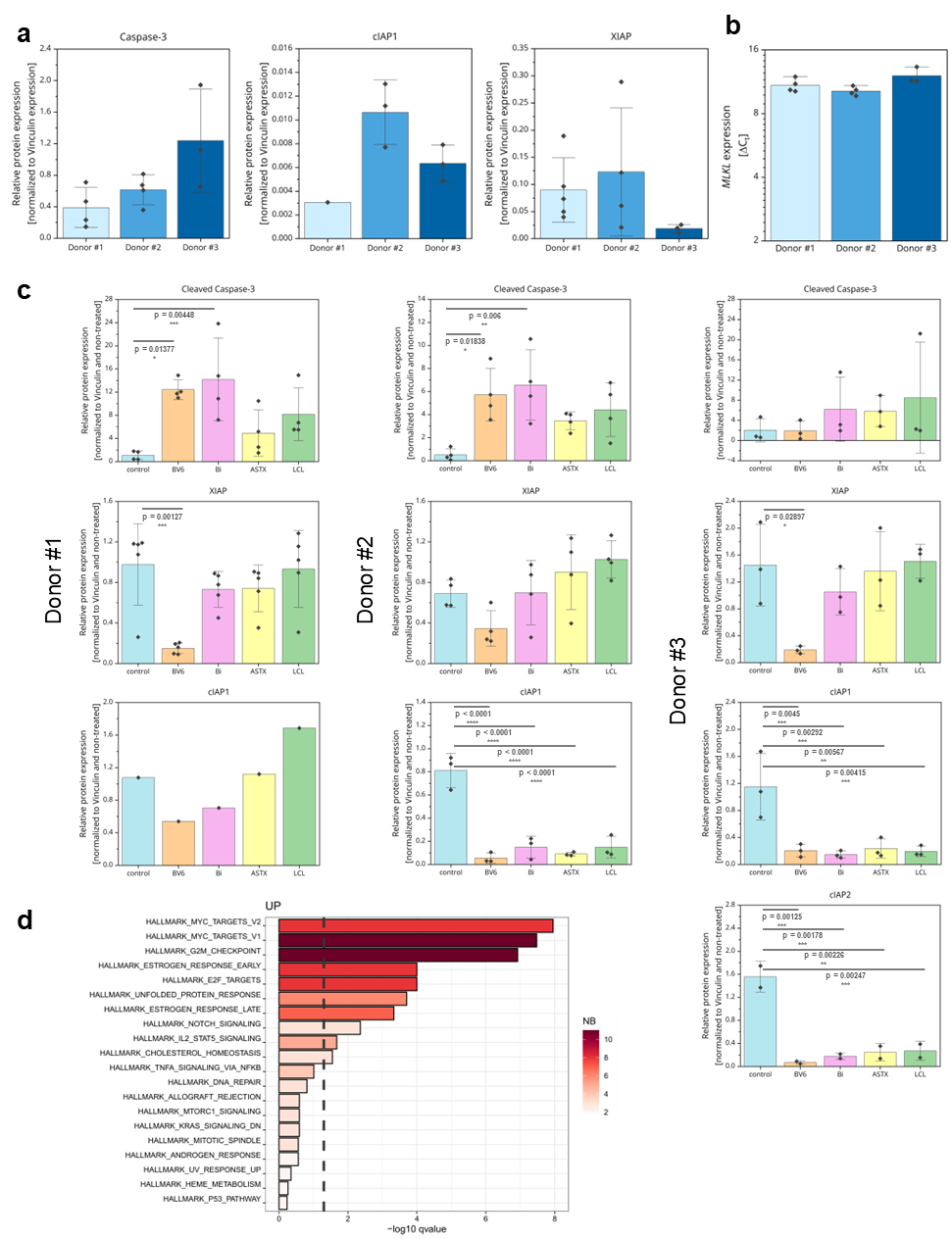
Supplementary Figure 4**

**Supplementary Fig. 4 related to Fig. 3: hMOs express proteins relevant for extrinsic apoptosis and treatment with Birinapant upregulates MYC signaling.** (**a**) Protein expression of caspase-3, cIAP1 and XIAP were assessed in Western blot and relative expression levels normalized to vinculin were calculated. (**b**) mRNA expression levels of *MLKL* in hMOs from donor #1, #2 and #3 normalized to reference genes *TBP*, *RPL13*, *RPII* and *18S-rRNA*. (**c**) Quantification of cIAP1, XIAP, cleaved caspase-3 and cIAP2 from Western blot (**Fig. 3a**) normalized to vinculin expression and non-treated controls. Error bars represent the standard deviation. One-way ANOVA followed by Tukey’s test was used to calculate statistical significance. **** p < 0.0001; *** p ≤ 0.005; ** p ≤ 0.01; * p ≤ 0.05; n.s. (not significant) p > 0.05. (**d**) GSEA and hallmark analysis were applied to hMOs from all three donors combined upon treatment with 10 µM Birinapant. The x-axis represents the statistical significance of enrichment (–log₁₀ adjusted q-value), and the y-axis lists the top enriched gene sets. Bars are color-coded according to the number of differentially expressed genes (DEGs) contained within each gene set. The dashed vertical line indicates the threshold for statistical significance (-log_10_ adjusted q-value(0.05)).

**Supplementary Figure 5**

**
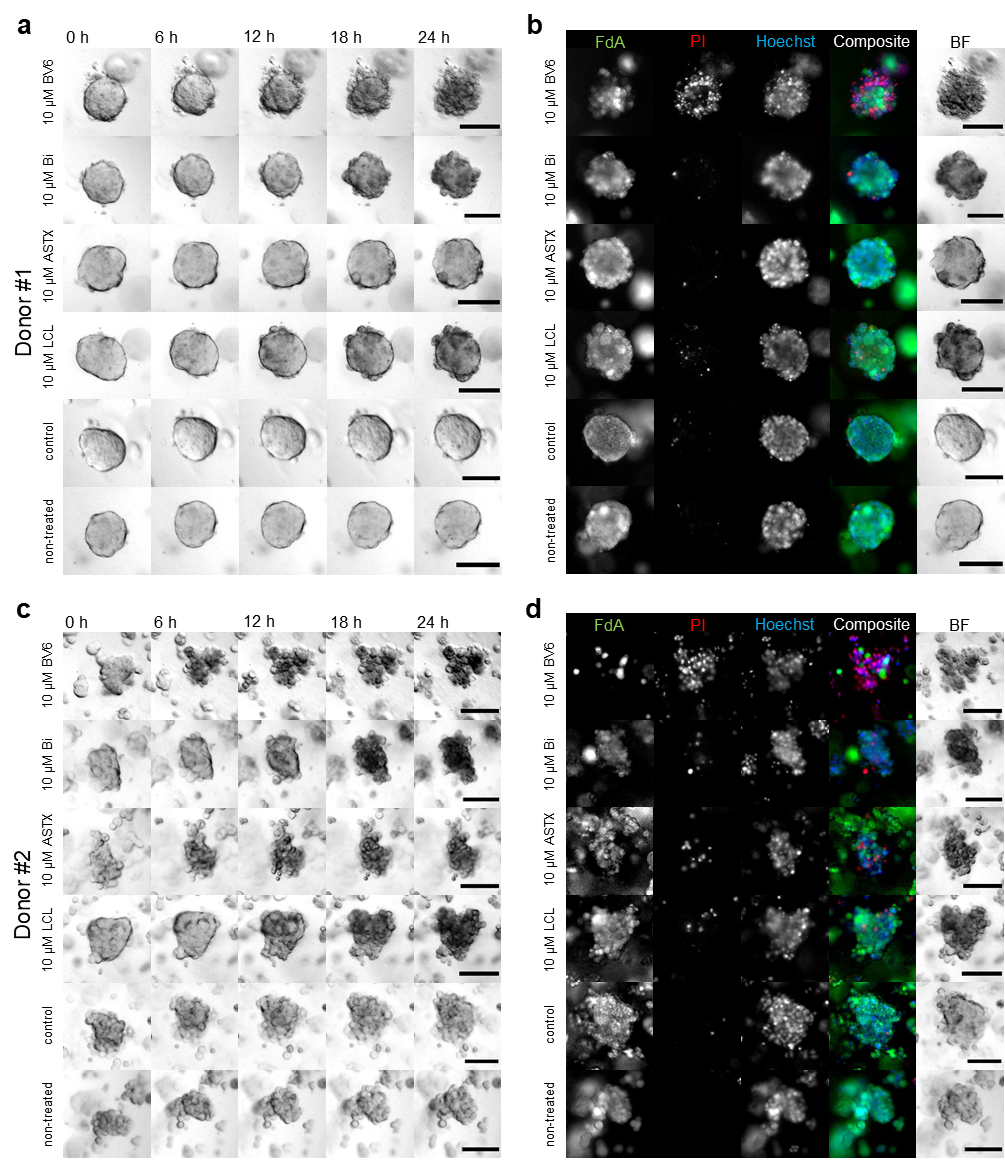
Supplementary Fig. 5 related to Fig. 3: Smac mimetics induce PCD in hMOs from donor #1 and #2.** hMOs from donor #1 (**a-b**) and #2 (**c-d**) were treated with 10 µM BV6, Birinapant (Bi), ASTX-660 (ASTX) or LCL-161 (LCL), control-treated (0.4 % DMSO) or non-treated and subjected to brightfield (BF) time-lapse microscopy for 24 h and shown after 0, 6, 12, 18 and 24 h of imaging. After 24 h live imaging, the same hMOs from (**a**) and (**c**) were stained using fluorescein diacetate (FdA, viable cells, green), propidium iodide (PI, dead cells, red) and Hoechst33342 (all nuclei, blue) and imaged again. Representative images of hMOs from donor #1 (**a-b**) and #2 (**c-d**), scale bars: 100 µm.

**Supplementary Figure 6**

**
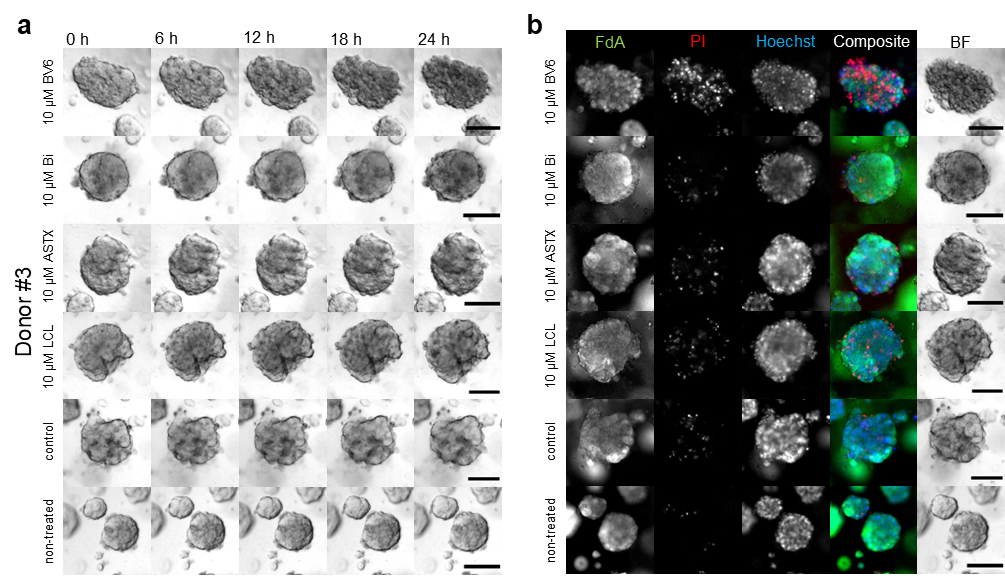
Supplementary Fig. 6 related to Fig. 3: Smac mimetics induce PCD in hMOs from donor #3.** (**a**) hMOs from donor #3 were treated with 10 µM BV6, Birinapant (Bi), ASTX-660 (ASTX) or LCL-161 (LCL), control-treated (0.4 % DMSO) or non-treated and subjected to brightfield (BF) time-lapse microscopy for 24 h and shown after 0, 6, 12, 18 and 24 h of imaging. (**b**) After 24 h live imaging, the same hMOs from (**a**) were stained using fluorescein diacetate (FdA, viable cells, green), propidium iodide (PI, dead cells, red) and Hoechst33342 (all nuclei, blue) and imaged again. Representative images of hMOs from donor #3, scale bars: 100 µm.

**Supplementary Figure 7**

**
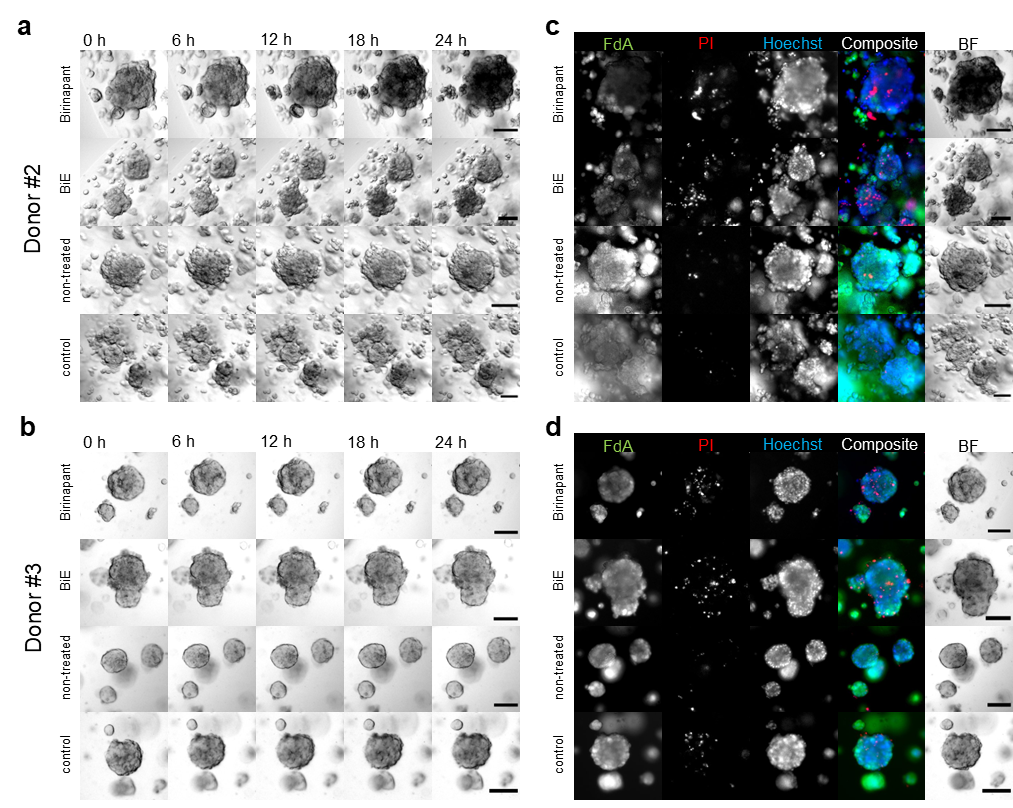
Supplementary Fig. 7 related to Fig. 4:** **Smac mimetics induce PCD in hMOs from donor #2 and #3.** (**a-b**) Brightfield (BF) time lapse images of BiE (10 µM Birinapant, 10 µM Emricasan)_-_, Bi (10 µM Birinapant)-, non- and control (1.5 % DMSO)-treated hMOs were acquired using the Zeiss Z1 Axioimager widefield microscope with 30 min intervals for 24 h and shown after 0, 6, 12, 18 and 24 h of imaging. (**c-d**) After 24 h live imaging, the same hMOs from (**a**) and (**b**) were stained using fluorescein diacetate (FdA, viable cells, green), propidium iodide (PI, dead cells, red) and Hoechst33342 (all nuclei, blue) and imaged again. Representative images of donor #2 (**a, c**) and #3 (**b, d**). Scale bars: 100 µm.

**Supplementary Figure 8**

**
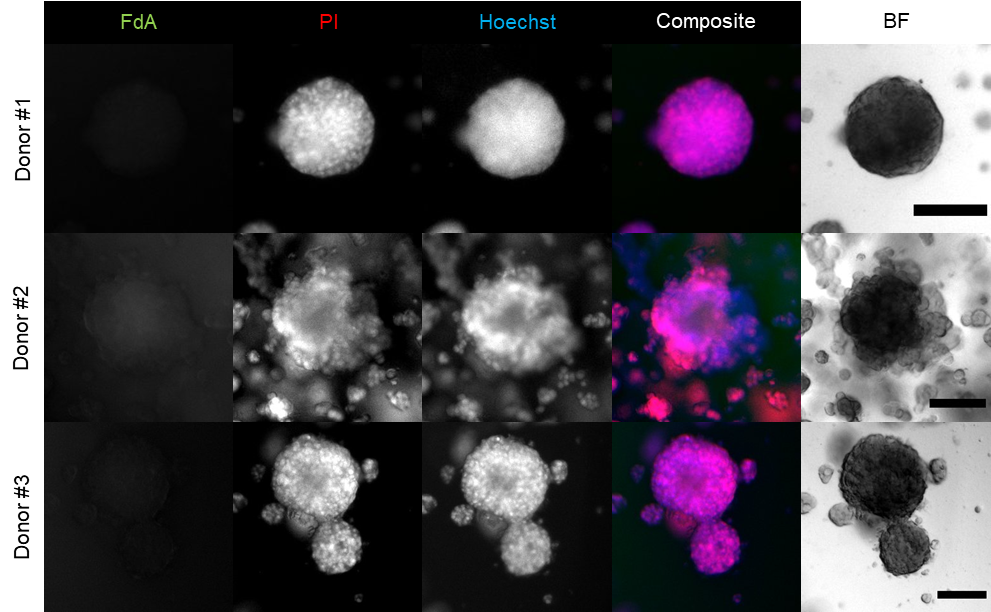
Supplementary Fig. 8 related to Fig. 4 and Supplementary Fig. 7: Propidium iodide successfully stains dead cells in hMOs from different donors.** hMOs from the same experiments as in **Fig. 4a-b** and **Supplementary Fig. 7** were killed by applying 100 % DMSO for 5 min and subsequently, a live-dead assay was performed using fluorescein diacetate (FdA, viable cells, green), propidium iodide (PI, dead cells, red) and Hoechst33342 (all nuclei, blue). Representative images of hMOs from donor #1, #2 and #3, scale bars: 100 µm.

**Supplementary Figure 9**

**
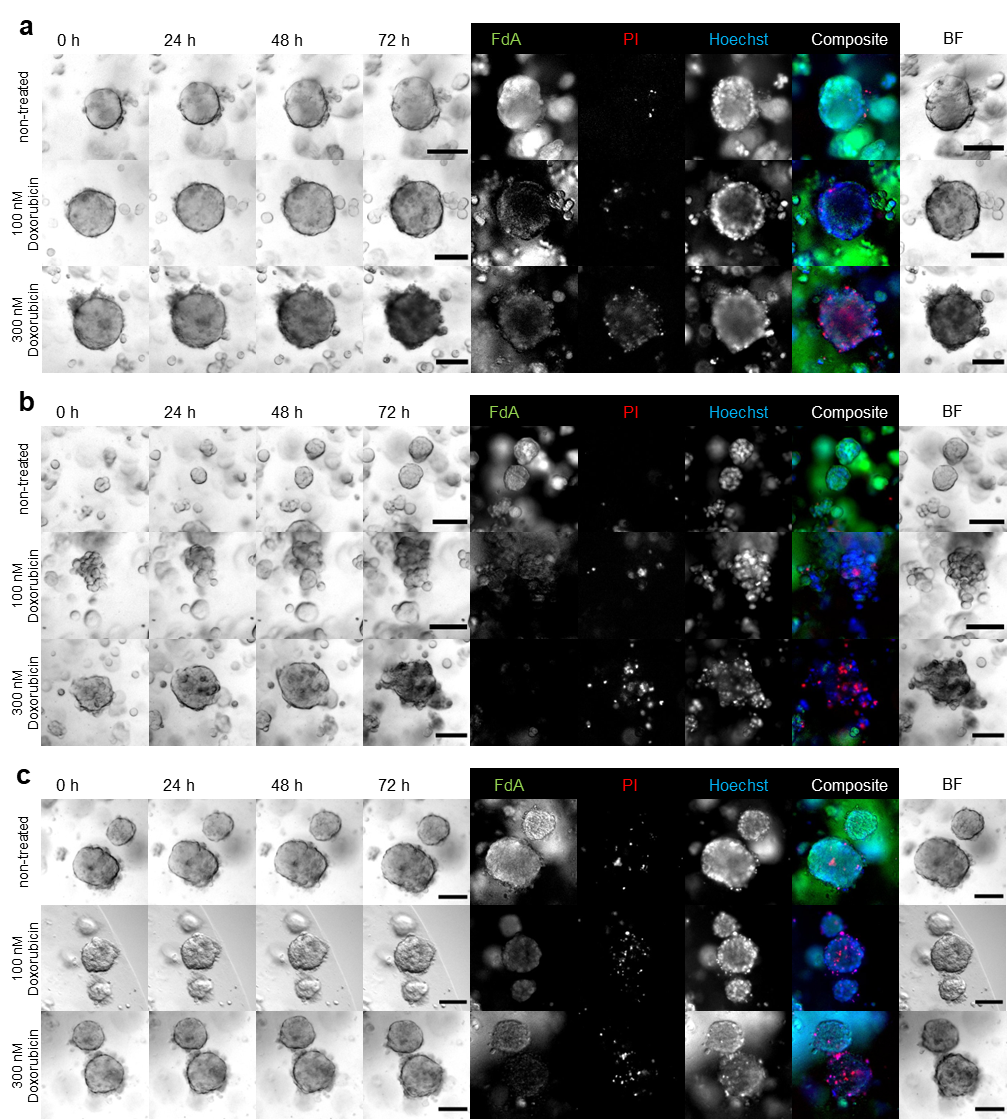
Supplementary Fig. 9 related to Fig. 4 and Supplementary Fig. 7: Induction of PCD by treatment with chemotherapeutic agent Doxorubicin in hMOs from different donors.** (**a-c**) Brightfield (BF) time lapse images of hMOs treated with 100 nM and 300 nM Doxorubicin or non-treated were acquired using the Zeiss Z1 Axioimager widefield microscope with 30 min intervals for 72 h and shown after 0, 24, 48 and 72 h of imaging. After 72 h live imaging, the same hMOs from (**a-c**) were stained with fluorescein diacetate (FdA, viable cells, green), propidium iodide (PI, dead cells, red) and Hoechst33342 (all nuclei, blue) and imaged again. Representative images of hMOs from donor #1 (**a**), #2 (**b**) and #3 (**c**), scale bars: 100µm.

**Supplementary Figure 10**

**
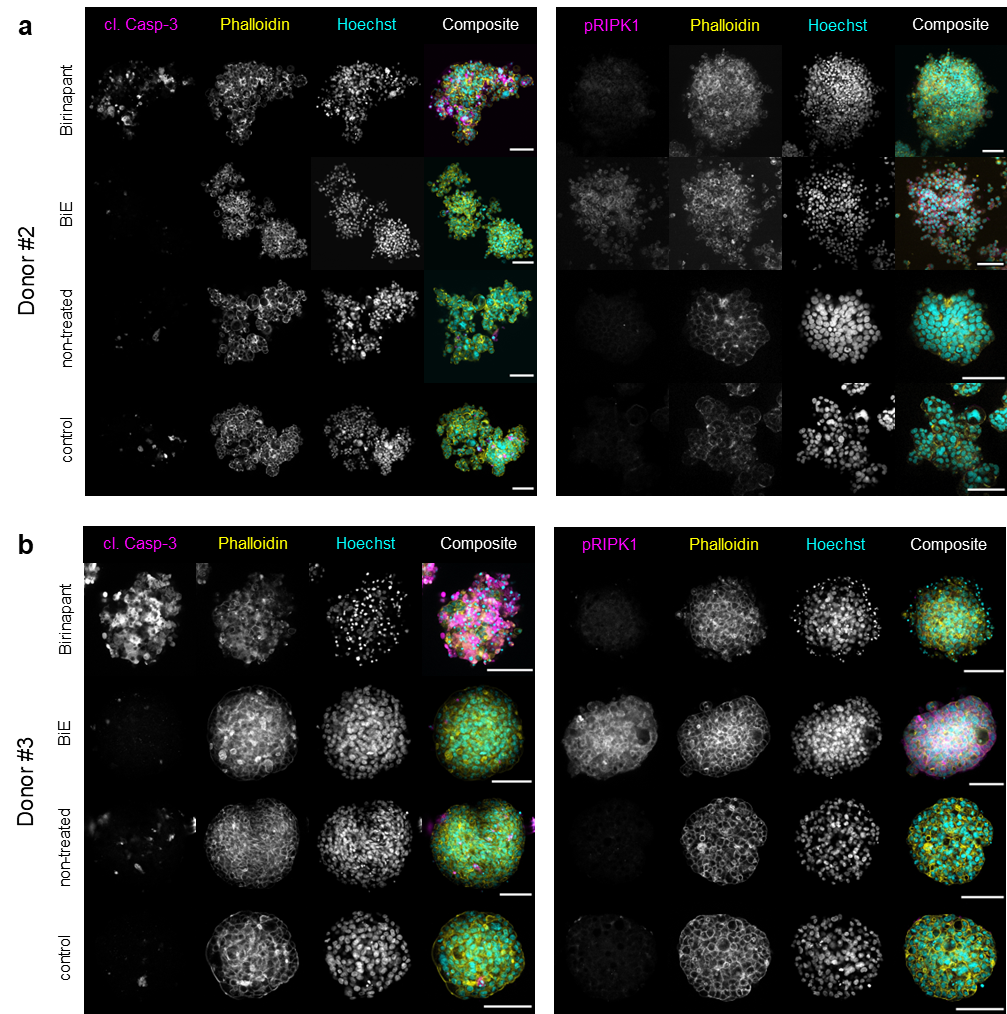
Supplementary Fig. 10 related to Fig. 4:** **Birinapant- and Emricasan-induced cell death shows hallmarks of apoptosis and necroptosis.** Immunofluorescence staining against cleaved caspase-3 (cl. Casp-3, magenta) and S166-phosphorylated RIPK1 (pRIPK1, magenta) and counterstained using AF647™-Phalloidin (yellow) and Hoechst33342 (cyan) in Birinapant-, BiE (10µM Birinapant, 10 µM Emricasan)-, non- and control (1.5 % DMSO)-treated hMOs after 24 h treatment from donor #2 (**a**) or #3 (**b**). Organoids were cleared with CUBIC-2 and imaged using a Zeiss LSM780 confocal microscope. Scale bars: 100 µm.

**Supplementary Figure 11**

**
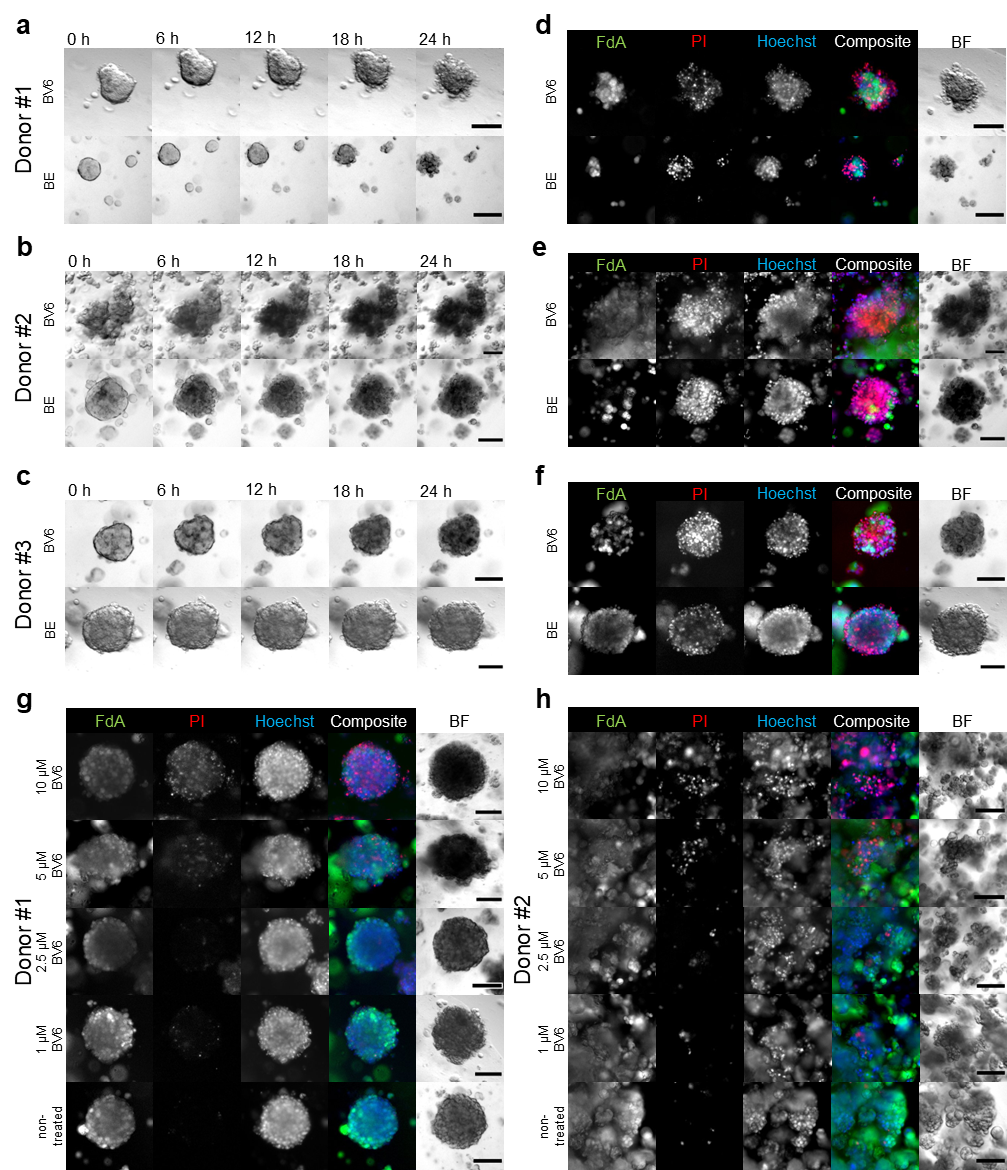
Supplementary Fig. 11 related to Fig. 4: Smac mimetic BV6 induces PCD in hMOs.** (**a-c**) Brightfield (BF) time-lapse imaging of hMOs treated with 10 µM BV6 or BE (10 µM BV6, 10 µM Emricasan) was performed using the Zeiss Z1 Axioimager widefield microscope with 30 min intervals for 24 h and shown after 0, 6, 12, 18 and 24 h of imaging. (**d-f**) After 24 h live imaging, the same hMOs from (**a-c**) were stained using fluorescein diacetate (FdA, viable cells, green), propidium iodide (PI, dead cells, red) and Hoechst33342 (all nuclei, blue) and imaged again. The corresponding controls can be found in **Fig. 4** and **Supplementary Fig. 7**. Representative images of hMOs from donors #1 (**a, d**), #2 (**b, e**) and #3 (**c, f**). (**g-h**) Live-dead assay following 24 h treatment with different concentrations of BV6 from donor #1 (**g**) and #2 (**h**). 10 µM BV6 was selected as final concentration moving forward. Scale bars: 100 µm.

**Supplementary Figure 12**

**
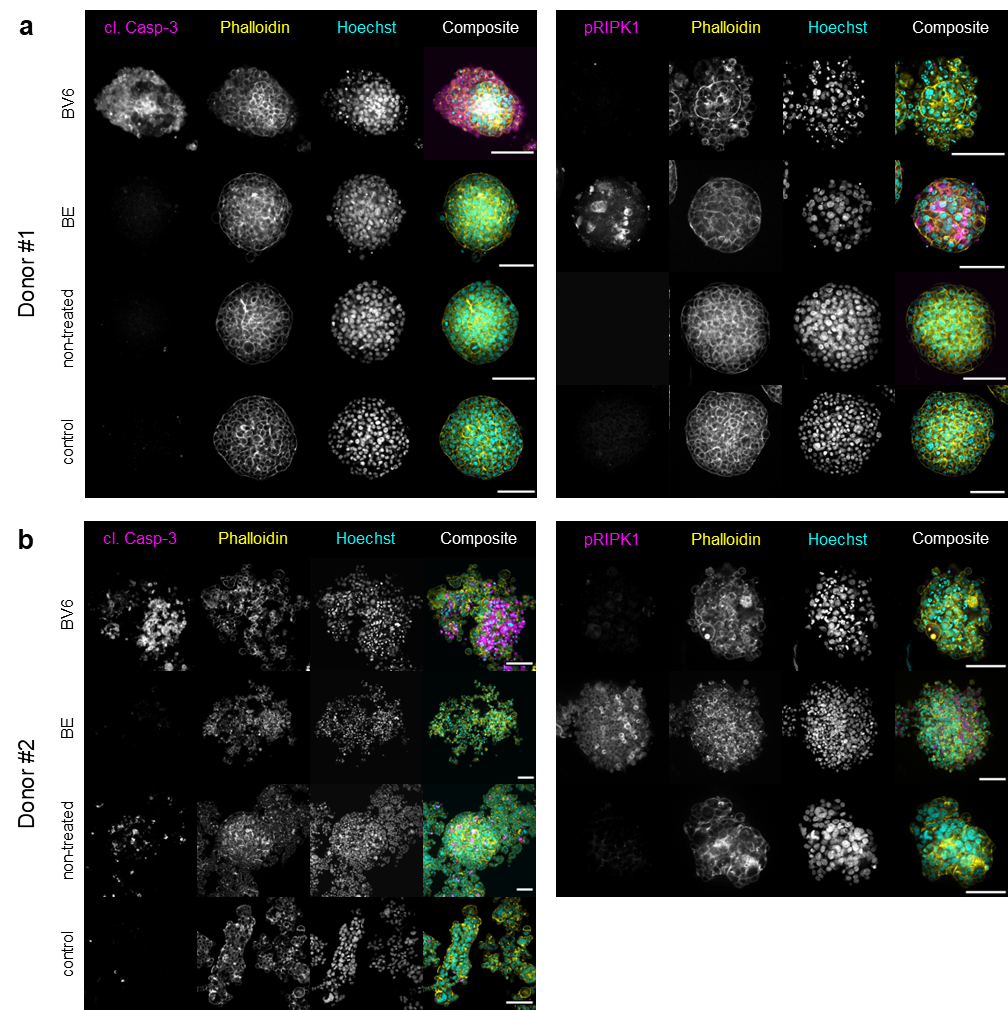
**

**
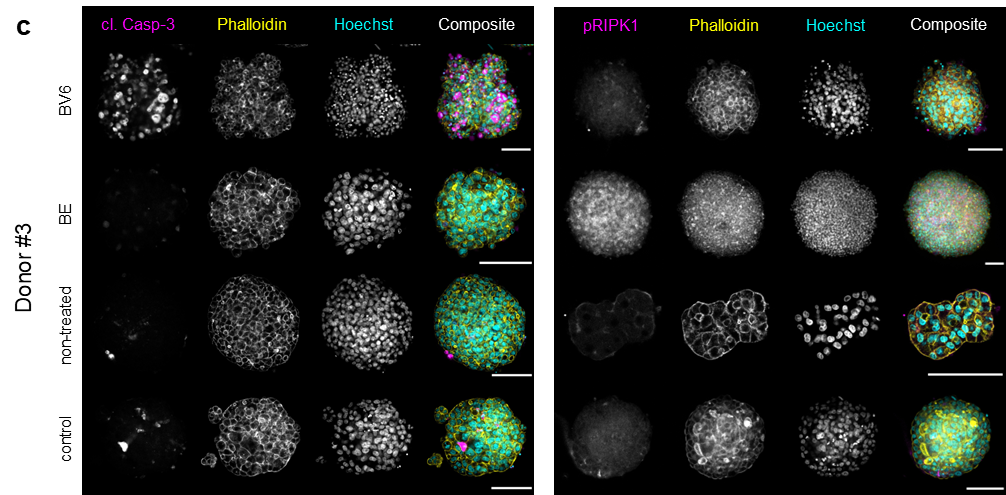
Supplementary Fig. 12 related to Fig. 4:** **Smac mimetic BV6 induces apoptosis and necroptosis.** Immunofluorescence staining against cleaved caspase-3 (cl. Casp-3, magenta) or S166-phosphorylated RIPK1 (pRIPK1, magenta) on hMOs after 24 h treatment with 10 µM BV6 or BE (10 µM BV6, 10 µM Emricasan). Organoids were counterstained using AF647™-Phalloidin (yellow) and Hoechst33342 (cyan) and cleared with CUBIC-2 prior to imaging with a Zeiss LSM780 confocal microscope. Representative images of hMOs from donors #1 (**a**), #2 (**b**) and #3 (**c**) are shown. Scale bars: 100 µm.

**Supplementary Figure 13**

**
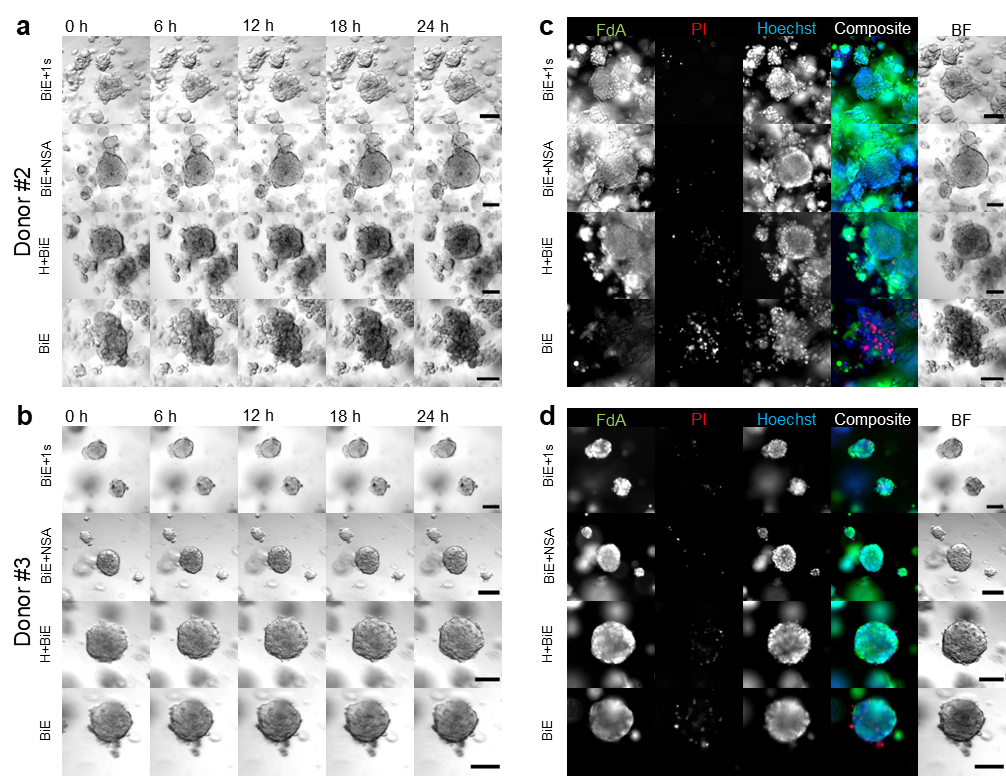
Supplementary Fig. 13 related to Fig. 5:** **Pharmacological modulation of LUBAC and necroptosis effectors rescue** **Birinapant- and Emricasan-induced PCD in hMOs from different donors.** (**a-b**) Brightfield (BF) time-lapse imaging was performed on BiE (10 µM Birinapant, 10 µM Emricasan)-treated hMOs co-treated with 1s (30 µM Necrostatin-1s), NSA (10 µM Necrosulfonamide) or pre-treated with H (30 µM HOIPIN-8) using the Zeiss Z1 Axioimager widefield microscope with 30 min intervals for 24 h and shown after 0, 6, 12, 18 and 24 h. (**c-d**) After 24 h live imaging, the same hMOs as in (**a-b**) were stained using fluorescein diacetate (FdA, viable cells, green), propidium iodide (PI, dead cells, red) and Hoechst33342 (all nuclei, blue) and imaged again. (**a-d**) Representative images from donor #2 (**a, c**) and #3 (**b, d**). Scale bars: 100 µm.

**Supplementary Figure 14**

**
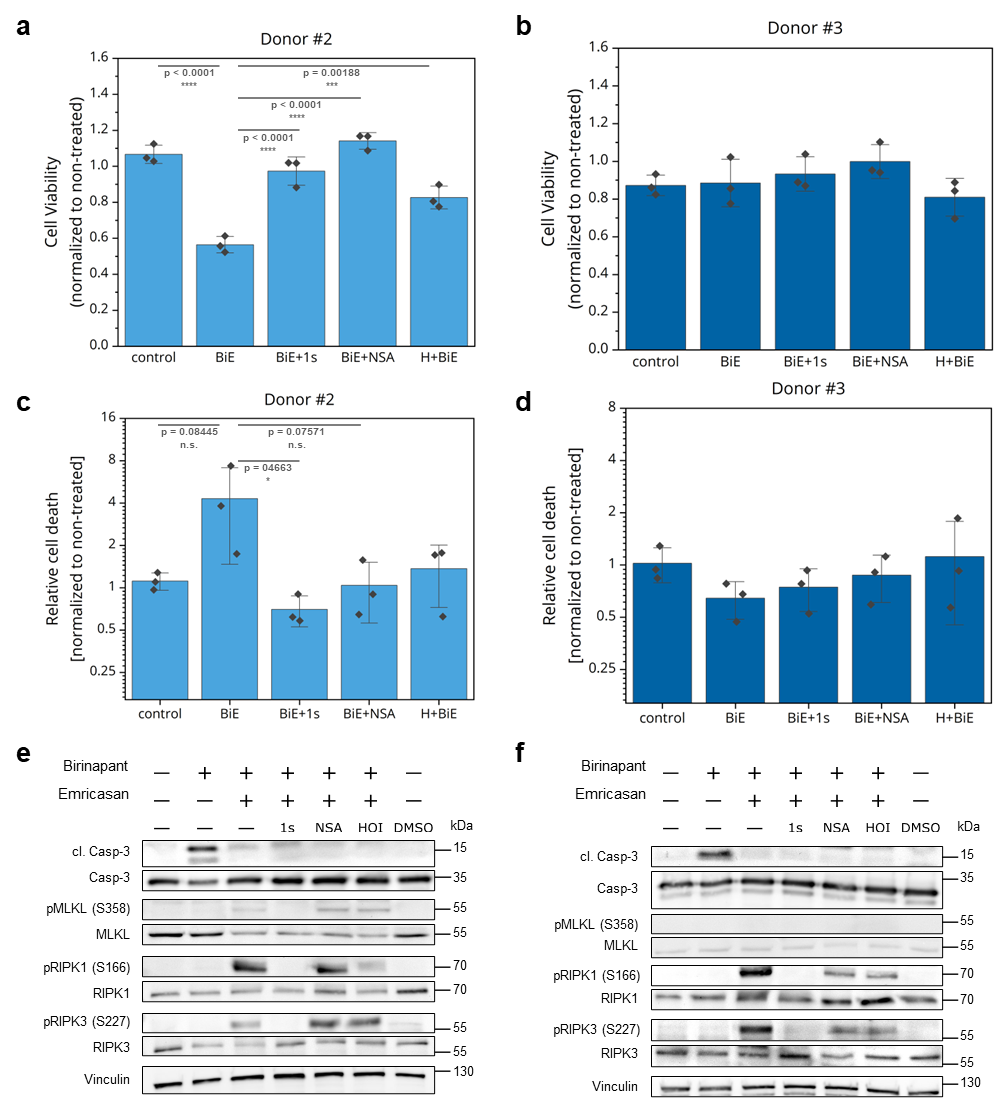
Supplementary Fig. 14 related to Fig. 5:** **Pharmacological modulation of LUBAC and necroptosis effectors rescue** **Birinapant- and Emricasan-induced cell viability, PCD and post-translational modifications in hMOs from different donors.** (**a-b**) CellTiter-Glo viability assays were performed on hMOs from donor #2 (**a**) and #3 (**b**) after 24 h treatment. Values were normalized to non-treated controls. One-way ANOVA followed by Tukey’s test was used to calculate statistical significance. **** p < 0.0001; *** p ≤ 0.005; ** p ≤ 0.01; * p ≤ 0.05; n.s. (not significant) p > 0.05. (**c-d**) Live-dead assays from donor #2 (**c**) and #3 (**d**) were quantified and normalized to non-treated controls. Error bars represent the standard deviation. One-way ANOVA followed by Tukey’s test was used to calculate statistical significance. **** p < 0.0001; *** p ≤ 0.005; ** p ≤ 0.01; * p ≤ 0.05; n.s. (not significant) p > 0.05. (**e-f**) Western blot analysis of cleaved and total caspase-3 (Casp-3) and phosphorylated and total RIPK1, RIPK3 and MLKL in treated hMOs. Vinculin served as loading control. Representative blots of donor #2 (**e**) and #3 (**f**) of at least two independent experiments are shown.

**Supplementary Figure 15**

**
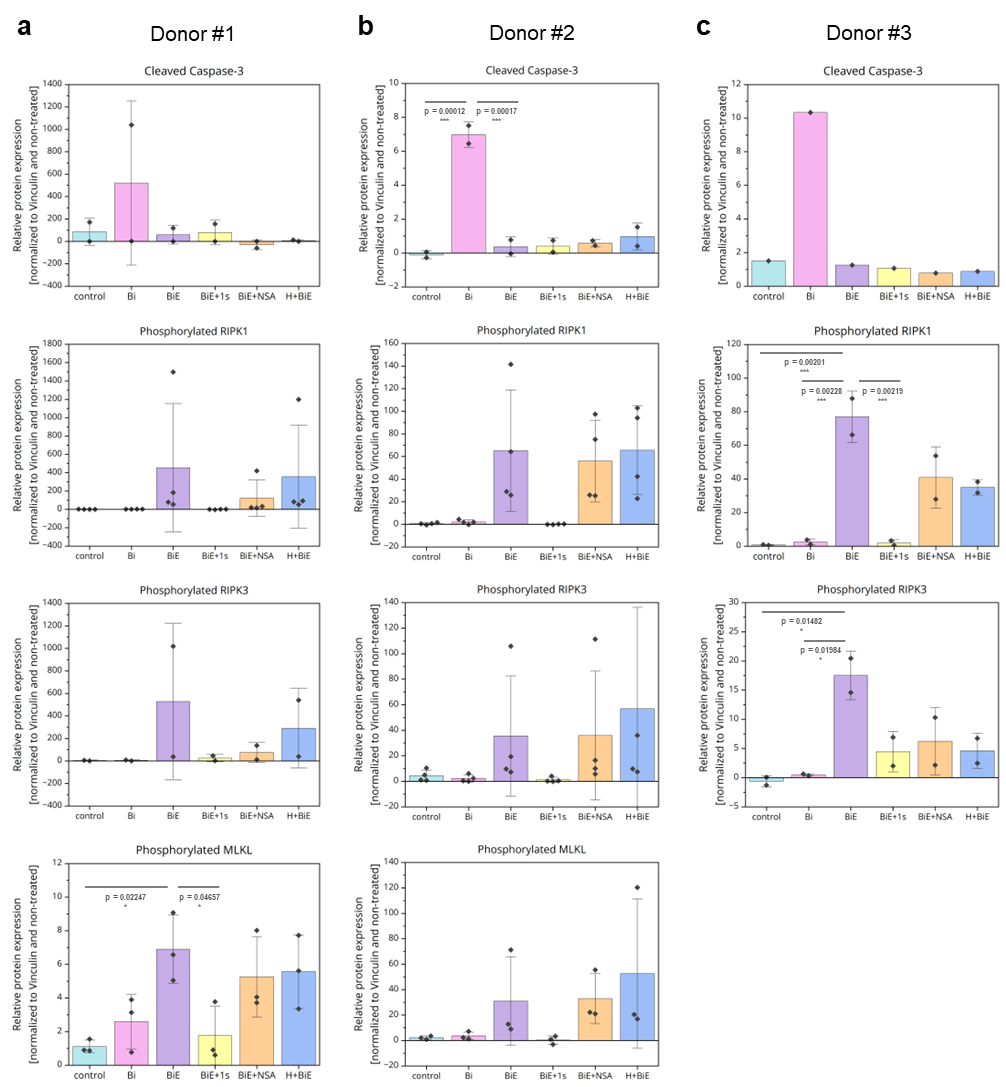
Supplementary Fig. 15 related to Fig. 5 and Supplementary Fig. 14: Treatment with Smac mimetics increase caspase-3 cleavage and phosphorylation of RIPK1, RIPK3 and MLKL in apoptotic and necroptotic hMOs, respectively.** Quantification of cleaved caspase-3, phosphorylated RIPK1, RIPK3 and MLKL from Western blot (**Fig. 5a** and **Supplementary Fig. 14e-f**) normalized to vinculin expression and non-treated controls. One-way ANOVA followed by Tukey’s test was used to calculate statistical significance. **** p < 0.0001; *** p ≤ 0.005; ** p ≤ 0.01; * p ≤ 0.05; n.s. (not significant) p > 0.05.

**Supplementary Figure 16**

**
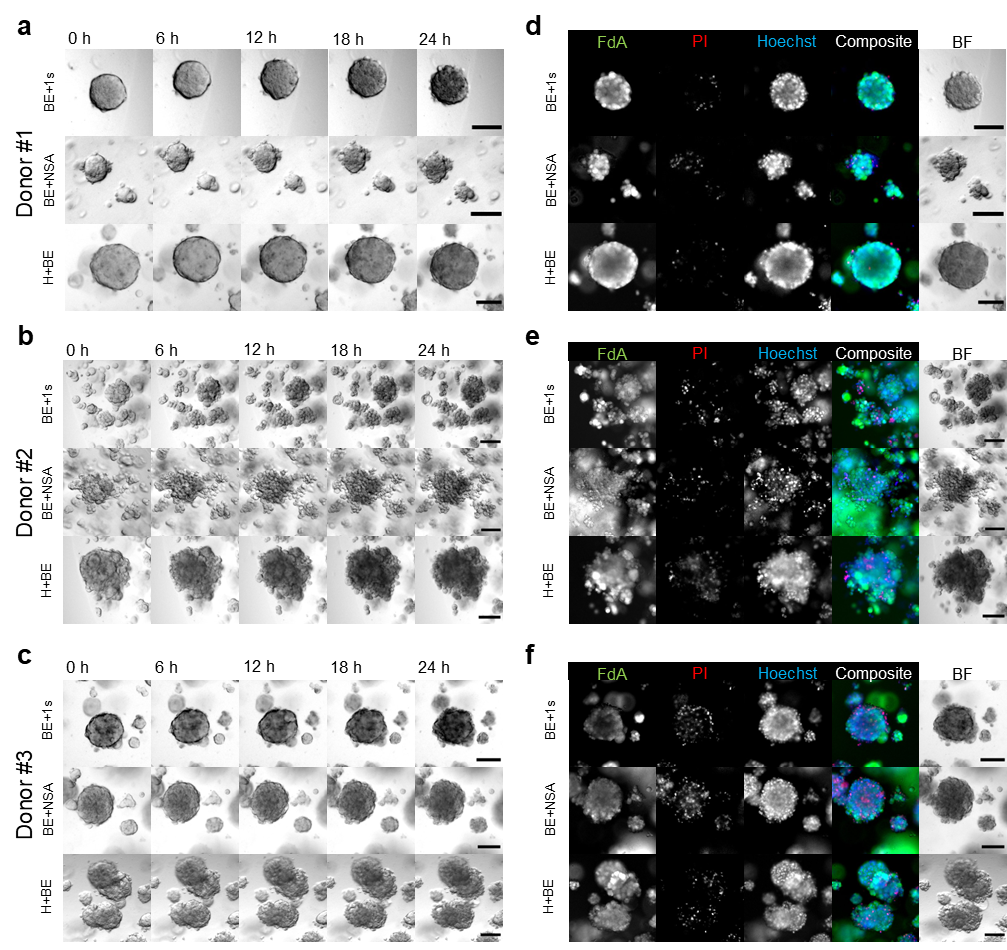
Supplementary Fig. 16 related to Fig. 5:** **Pharmacological modulation of LUBAC and necroptosis effectors rescue BV6- and Emricasan-induced PCD in hMOs from all donors.** (**a-c**) Brightfield (BF) time-lapse imaging was performed on BE (10 µM BV6, 10 µM Emricasan)-treated hMOs co-treated with 1s (30 µM Necrostatin-1s), NSA (10 µM Necrosulfonamide) or pre-treated with H (30 µM HOIPIN-8) using the Zeiss Z1 Axioimager widefield microscope with 30 min intervals for 24 h and shown after 0, 6, 12, 18 and 24 h. (**d-f**) After 24 h live imaging, the same hMOs as in (**a-c**) were stained using fluorescein-diacetate (FdA, viable cells, green), propidium iodide (PI, dead cells, red) and Hoechst33342 (all nuclei, blue) and imaged again. (**a-f**) Representative images of hMOs from donors #1 (**a, d**), #2 (**b, e**) and #3 (**c, f**) are shown. Corresponding controls can be found in **Fig. 5** and **Supplementary Fig. 7**. Scale bars: 100 µm.

**Supplementary Figure 17**

**
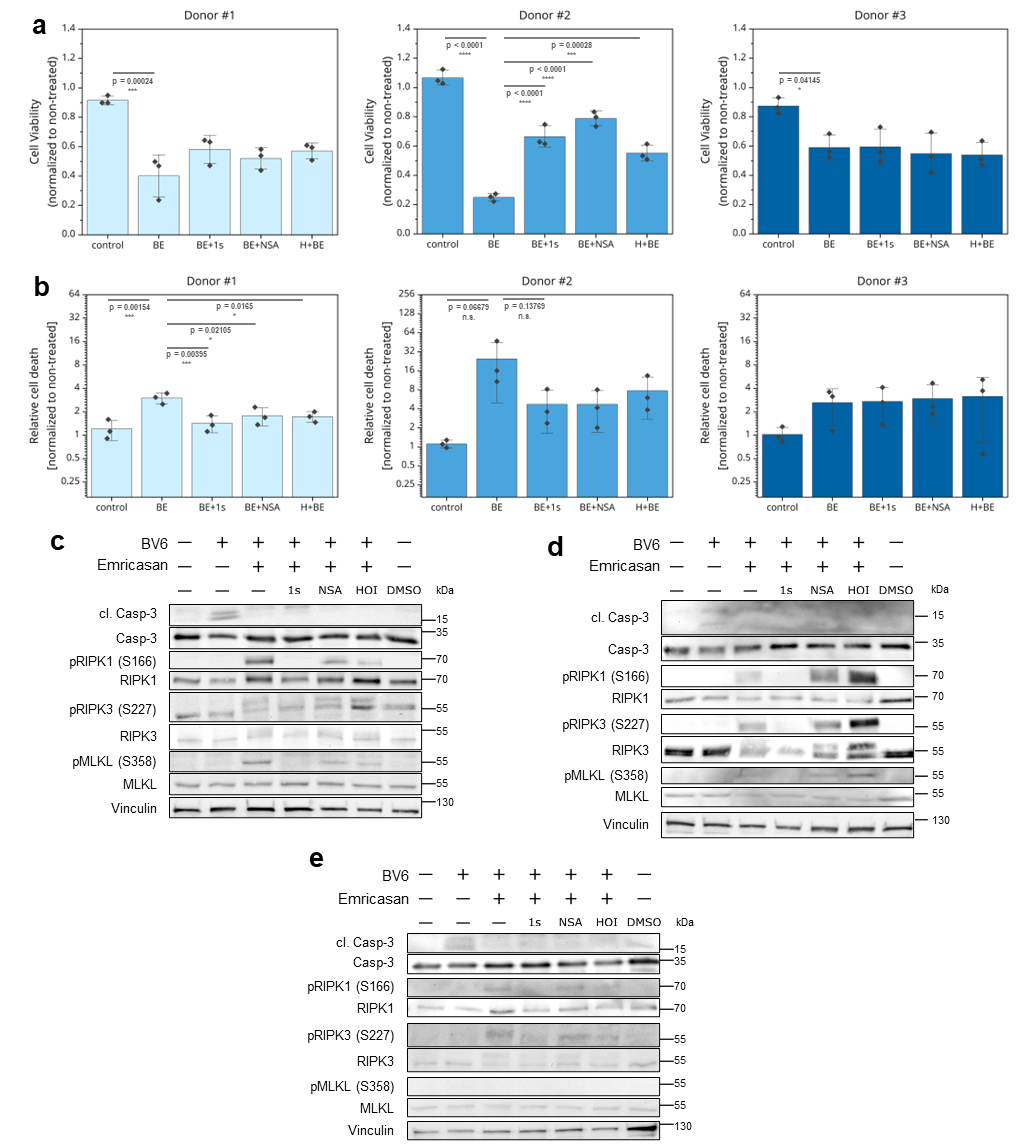
Supplementary Fig. 17 related to Fig. 5: Pharmacological modulation of LUBAC and necroptosis effectors rescue cell viability and phosphorylation in hMOs from all donors**. (**a**) Cell viability assay using Cell-Titer Glo of hMOs from donor #1, #2 or #3 upon treatment with BE (10 µM BV6, 10 µM Emricasan) and co-treated with 1s (30 µM Necrostatin-1s), NSA (10 µM Necrosulfonamide) or pre-treated with H (30 µM HOIPIN-8) and control-treated (1.5 % DMSO) as indicated for 24 h. Values were normalized to non-treated organoids. Error bars represent the standard deviation. One-way ANOVA followed by Tukey’s test was used to calculate statistical significance. **** p < 0.0001; *** p ≤ 0.005; ** p ≤ 0.01; * p ≤ 0.05; n.s. (not significant) p > 0.05. (**b**) Live-dead assays were quantified and normalized to non-treated controls of hMOs from donor #1, #2 and #3 treated as in (**a**). Error bars represent the standard deviation. One-way ANOVA followed by Tukey’s test was used to calculate statistical significance. **** p < 0.0001; *** p ≤ 0.005; ** p ≤ 0.01; * p ≤ 0.05; n.s. (not significant) p > 0.05. (**c-e**) Western blot analysis of cleaved and total caspase-3 (Casp-3) and phosphorylated and total RIPK1, RIPK3 and MLKL of treated hMOs as described previously. Vinculin served as loading control. Representative blots of donor #1 (**d**), #2 (**e**) and #3 (**f**) of at least two independent experiments are shown.

**Supplementary Figure 18**

**
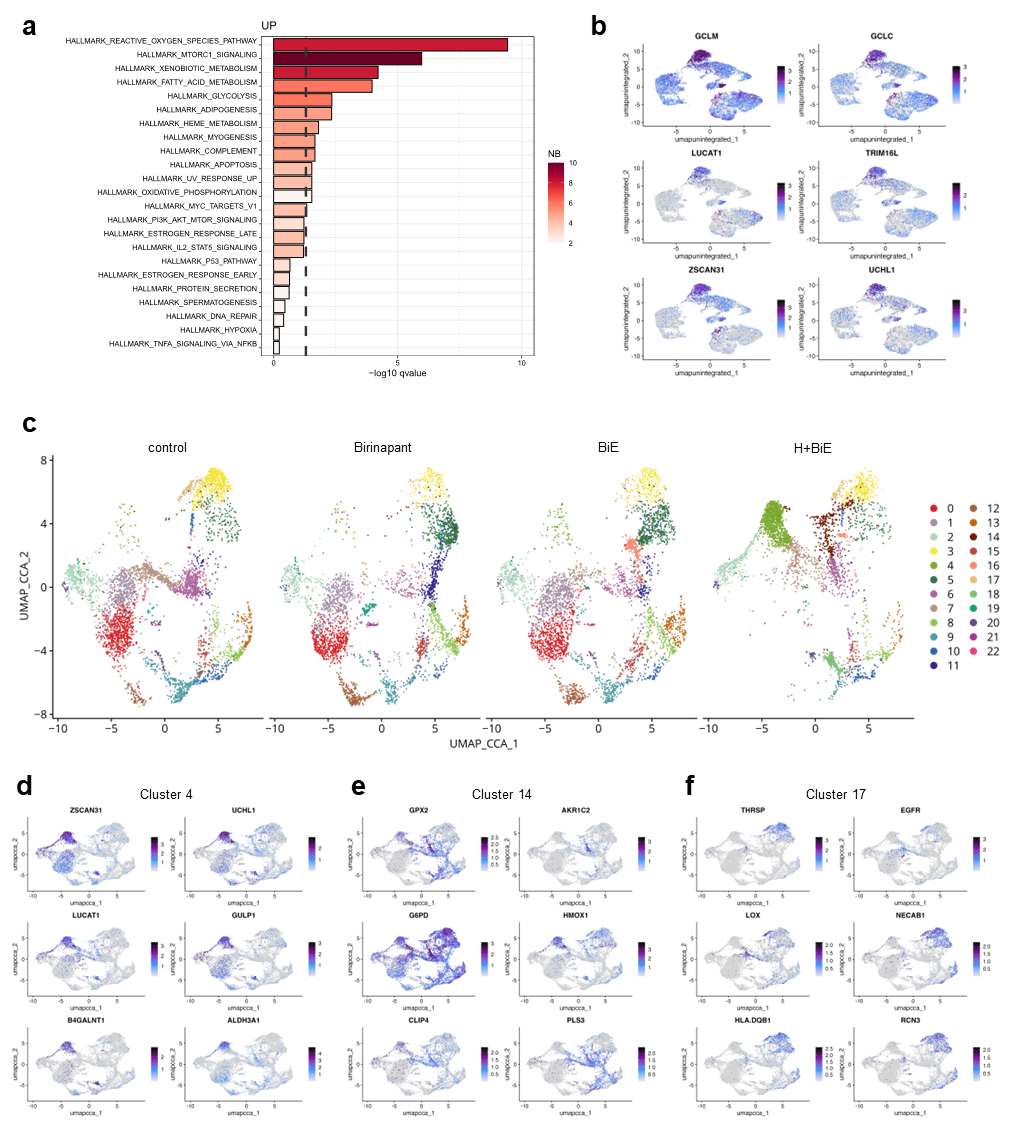
Supplementary Fig. 18 related to Fig. 5:** **LUBAC inhibition in necroptotic hMOs leads to induction of ROS signaling and fatty acid metabolism.** (**a**) Hallmark analysis of GSEA data of hMOs upon pre-treatment with 30 µM HOIPIN-8 and 24 h treatment with 10 µM Birinapant and 10 µM Emricasan of all donors combined. The x-axis represents the statistical significance of enrichment (–log₁₀ adjusted q-value), and the y-axis lists the top enriched gene sets. Bars are color-coded according to the number of differentially expressed genes (DEGs) contained within each gene set. The dashed vertical line indicates the threshold for statistical significance (-log_10_ adjusted q-value(0.05)). (**b**) UMAP of the six most upregulated genes in the hMOs as described before. (**c**) UMAP showing the 22 clusters per condition with all donors combined. hMOs were control-treated (1.5 % DMSO), Birinapant- (10 µM Birinapant), BiE- (10 µM Birinapant, 10 µM Emricasan) or H+BiE- (30 µM HOIPIN-8 pre-treatment, 10 µM Birinapant, 10 µM Emricasan) treated for 24 h. Each cluster is presented in a different color. (**d-f**) UMAP of the six most upregulated genes in clusters 4 (**d**), 14 (**e**) and 17 (**f**).

**Supplementary Figure 19**

**
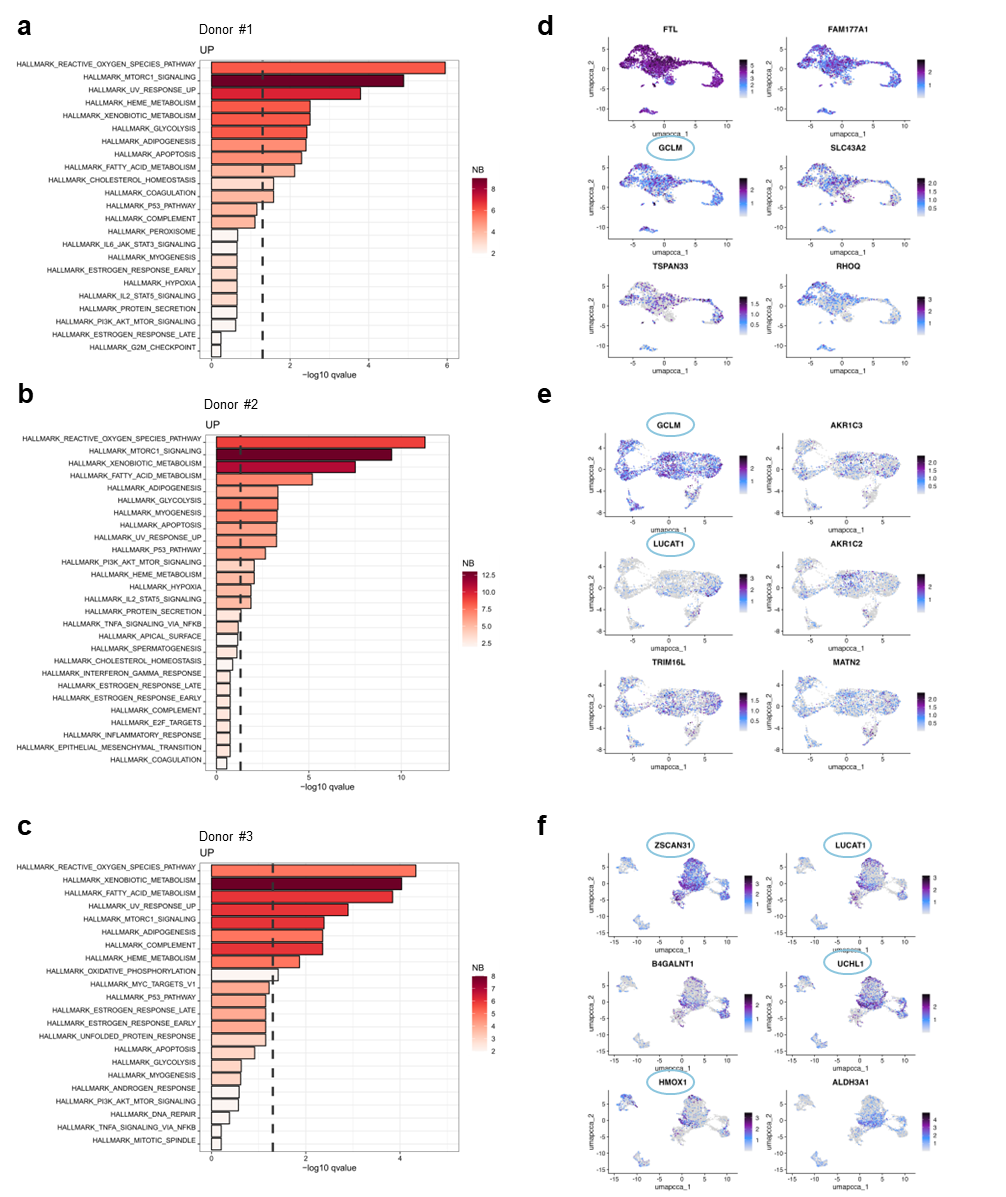
Supplementary Fig. 19 related to Fig. 5: LUBAC inhibition in necroptotic hMOs from individual donors confirms the induction of ROS signaling and changes in metabolism.** (**a-c**) Hallmark analysis of GSEA data of hMOs upon pre-treatment with 30 µM HOIPIN-8 and 24 h treatment with 10 µM Birinapant and 10 µM Emricasan of donor #1 (**a**), #2 (**b**) and #3 (**c**). The x-axis represents the statistical significance of enrichment (–log₁₀ adjusted q-value), and the y-axis lists the top enriched gene sets. Bars are color-coded according to the number of differentially expressed genes (DEGs) contained within each gene set. The dashed vertical line indicates the threshold for statistical significance (-log_10_ adjusted q-value(0.05)). (**d-f**) UMAP of the six most upregulated genes as described before in the hMOs from donor #1 (**d**), #2 (**e**) and #3 (**f**).

**
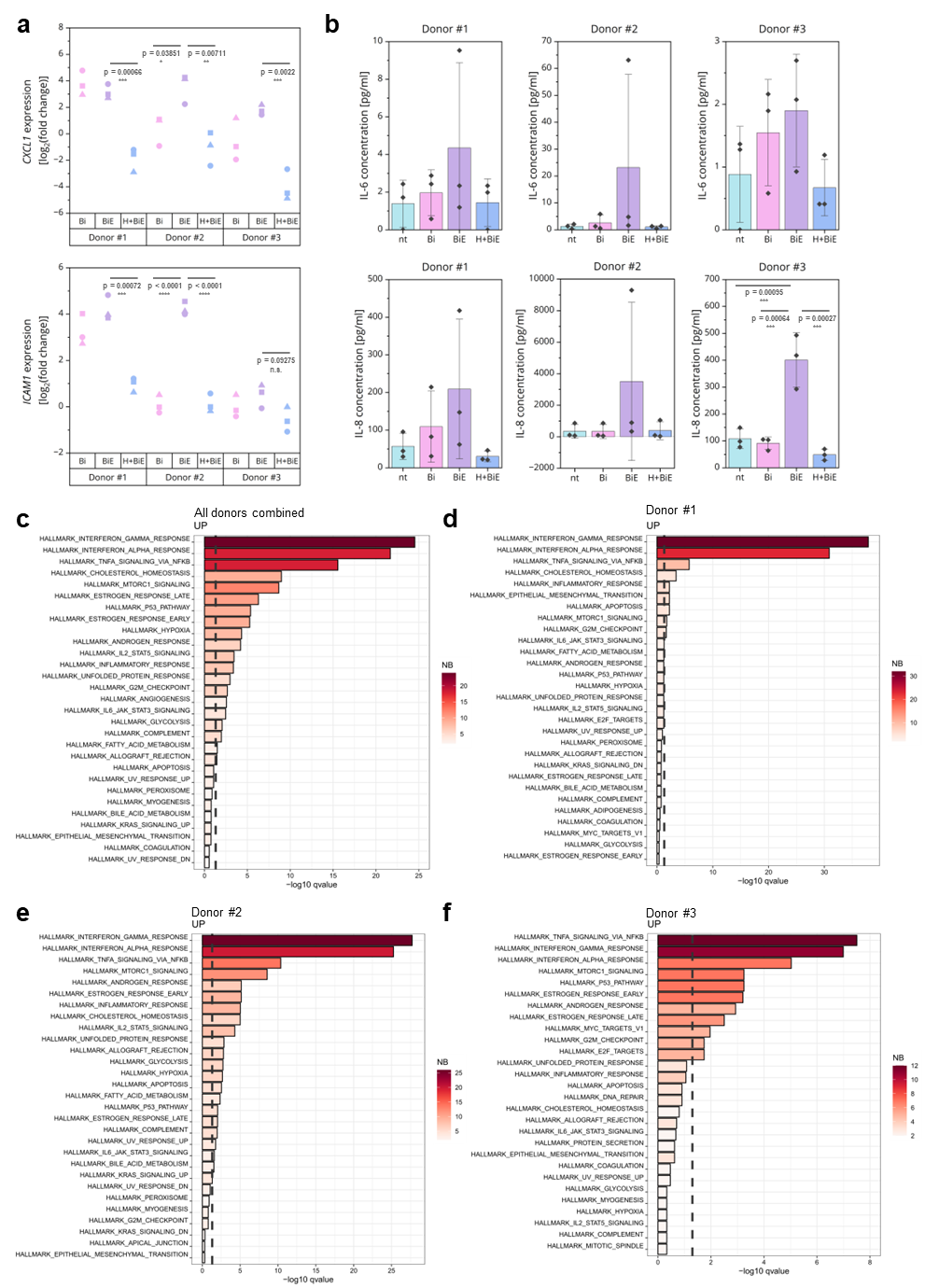
Supplementary Figure 20**

**Supplementary Fig. 20 related to Fig. 6: Necroptosis induces the secretion of inflammatory mediators in metastatic hMOs donor-independently.** (**a**) mRNA expression levels of *CXCL1* and *ICAM1* of HOIPIN-8 (30 µM) pre-treated hMOs upon treatment with 10 µM Birinapant (Bi) and 10 µM Emricasan (E) for 24 h. Gene expression was normalized to *RPII*, *18S-rRNA*, *TBP* and *RPL13* mRNA expression and is presented as log_2_(fold change). N=3 independent experiments are shown. One-way ANOVA followed by Tukey’s test was used to calculate statistical significance. **** p < 0.0001; *** p ≤ 0.005; ** p ≤ 0.01; * p ≤ 0.05; n.s. (not significant) p > 0.05. (**b**) TNF-α and IP-10 concentration in the supernatant from non-treated and treated hMOs measured by FACS-based CBA assay. Treatment was performed as in (**a**). N=3 independent experiments are shown. One-way ANOVA followed by Tukey’s test was used to calculate statistical significance. **** p < 0.0001; *** p ≤ 0.005; ** p ≤ 0.01; * p ≤ 0.05; n.s. (not significant) p > 0.05. (**c**) Hallmark analysis of the scCITEseq dataset using GSEA of all donors combined upon BiE-treatment for 24 h. (**d-f**) Complete list of hallmark analysis of the scCITEseq dataset using GSEA per donor upon BiE-treatment for 24 h from donor #1 (**d**), #2 (**e**) and #3 (**f**). (**c-f**) The x-axis represents the statistical significance of enrichment (–log₁₀ adjusted q-value), and the y-axis lists the top enriched gene sets. Bars are color-coded according to the number of differentially expressed genes (DEGs) contained within each gene set. The dashed vertical line indicates the threshold for statistical significance (-log_10_ adjusted q-value(0.05)).

**Supplementary Figure 21**

**
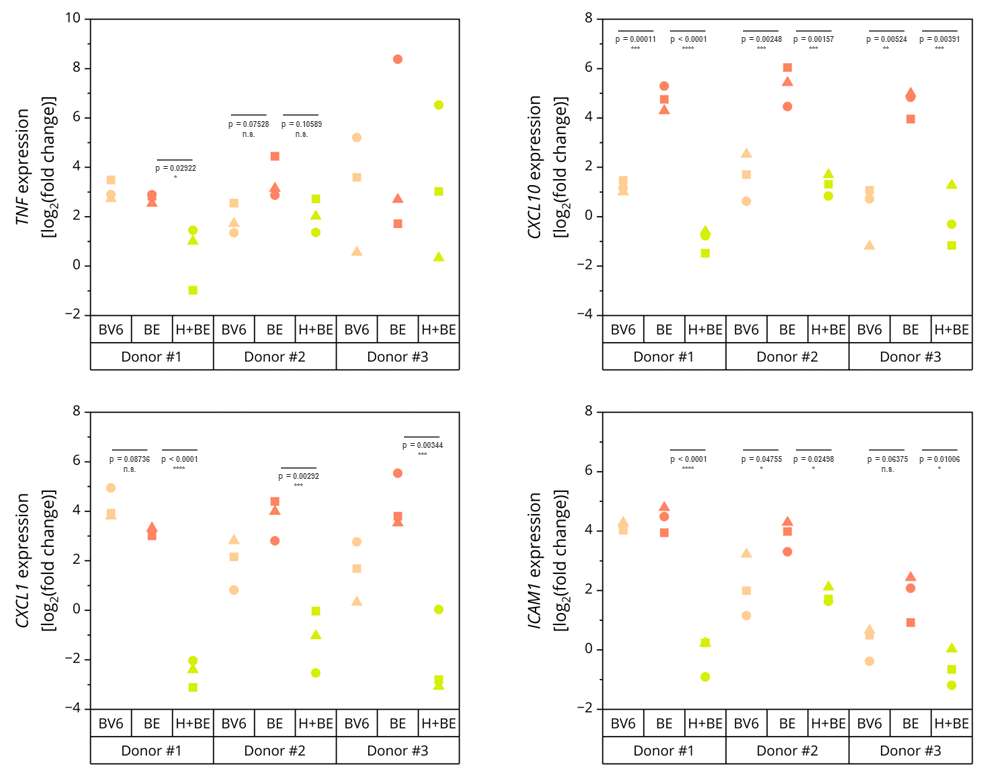
Supplementary Fig. 21 related to Fig. 6: BV6- and Emricasan-induced necroptosis induces inflammatory signaling that can be reversed by co-treatment with HOIPIN-8 in hMOs.** mRNA expression levels of *TNF*, *CXCL10*, *CXCL1* and *ICAM1* in hMOs treated with BV6 (10 µM BV6), BE (10 µM BV6, 10 µM Emricasan) or H+BE (30 µM HOIPIN-8 pre-treatment, 10 µM BV6, 10 µM Emricasan) for 24 h. Gene expression was normalized against non-treated conditions and *RPII, 18S-rRNA, TBP* and *RPL13* mRNA expression and is presented as log_2_(fold change).

**Supplementary Table 1: Medium composition for organoid growth medium.**

| **Compound** | **Type I organoid medium** | **Type II organoid medium** |
| --- | --- | --- |
|  | Advanced Dulbecco’s Modified Eagles Medium with Nutrient Mixture F-12 | Advanced Dulbecco’s Modified Eagles Medium with Nutrient Mixture F-12 |
| HEPES | 1M | 1 M |
| GlutaMAX | 1x | 1x |
| Penicillin/streptomycin | 100 U/ml | 100 U/ml |
| N-acetylcysteine | 1.25 mM | 1.25 mM |
| A83-01 | 500 nM | 500 nM |
| B27 without vitamin A | 1x | 1x |
| Y-27632 | 5 µM | 10 µM |
| Nicotinamide | 10 mM | 10 mM |
| Recombinant Noggin-fc fusion protein | 100 ng/ml | 100 ng/ml |
| R-spondin-3 conditioned medium | 250 ng/ml R-spondin-3 | - |
| Recombinant R-spondin-3 | - | 250 ng/ml |
| Human EGF | 5 ng/ml | 500 ng/ml |
| Primocin | 50 µg/ml | 50 µg/ml |
| FGF-7 | 5 ng/ml | 5 ng/ml |
| Heregulin-β | 37.5 ng/ml | 37.5 ng/ml |
| FGF-10 | 20 ng/ml | 40 ng/ml |
| SB202190 | 500 nM | - |
| Hydrocortisone | - | 500 ng/ml |
| Forskolin | - | 10 µM |
| β-estradiol | - | 368 nM |
| Wnt surrogate-fc fusion protein | - | 0.25 nM |

**Supplementary Table 2: List of primary antibodies.**

| **Primary antibody** | **Manufacturer** | **Article number** | **Application** |
| --- | --- | --- | --- |
| Caspase-3 | CST | 9662 | WB |
| CD49f | ThermoFisher Scientific | 710209 | IF |
| cIAP1 | R&D | AF8181 | WB |
| cIAP2 | CST | 3130 | WB |
| Cleaved caspase-3 | CST | 9661 | WB, IF |
| E-cadherin | CST | 14472 | IF |
| GATA-3 | abcam | ab199438 | IF |
| Ki67 | abcam | ab16667 | IF |
| Ki67 | CST | 9449 | IF |
| MLKL | CST | 14993 | WB |
| Phospho-MLKL S358 | CST | 91689 | WB |
| Phospho-RIPK1 S166 | CST | 657465 | WB, IF |
| Phospho-RIPK3 S227 | abcam | ab209384 | WB |
| RIPK1 | BD | 610459 | WB |
| RIPK3 | CST | 13526 | WB |
| Vinculin | Merck | V9131 | WB |
| XIAP | BD | 610716 | WB |

**Supplementary Table 3: List of secondary antibodies**

| **Secondary antibody** | **Manufacturer** | **Article number** | **Application** |
| --- | --- | --- | --- |
| donkey α-goat-HRP | Jackson Immuno Research | 705-035-147 | WB |
| goat α-mouse-HRP | Jackson Immuno Research | 115-035-003 | WB |
| goat α-rabbit-HRP | Jackson Immuno Research | 111-035-003 | WB |
| donkey α-mouse-AlexaFluor™568 | ThermoFisher Scientific | A10037 | IF |
| donkey α-rabbit-AlexaFluor™488 | ThermoFisher Scientific | A21206 | IF |
| donkey α-rabbit-AlexaFluor™647 | ThermoFisher Scientific | A32795 | IF |

**Supplementary Table 4: List of primers for RT-qPCR.**

| **Gene of interest** | **Forward primer** | **Reverse primer** |
| --- | --- | --- |
| 18S-rRNA | CGCAAATTACCCACTCCCG | TTCCAATTACAGGGCCTCGAA |
| CXCL1 | AACCGAAGTCATAGCCACAC | GTTGGATTTGTCACTGTTCAGC |
| CXCL10 | CTGAGCCTACAGCAGAGGAAC | GATGCAGGTACAGCGTACAGT |
| GZMB | CGTGGGGGACCCAGAGATTA | TTTCATTACAGCGGGGGCTT |
| ICAM1 | CTTCCTCACCGTGTACTGGAC | GGCAGCGTAGGGTAAGGTTC |
| IFIT1 | CTTGTGGGTAATACAGTGGAGATG | GCTCCAGACTATCCTTGACCTG |
| IFNG | GGCATTTTGAAGAATTGGAAAG | TTTGGATGCTCTGGTCATCTT |
| IRF9 | AGCCTGGACAGCAACTCAG | GAAACTGCCCACTCTCCACT |
| ISG15 | GAGGCAGCGAACTCATCTTT | AGCATCTTCACCGTCAGGTC |
| NCR1 | GCCAGGGAAATTATGGGGCT | CAGGATGAACCGAGAGGGTG |
| PRF1 | GTGGAGTGCCGCTTCTACAG | TGCCGTAGTTGGAGATAAGCC |
| RPII | GCACCACGTCCAATGACAT | GTGCGGCTGCTTCCATAA |
| RPL13 | AAGATCCGCAGACGTAAGGC | GGACTCCGTGGACTTGTTCC |
| TBP | TAAGAGAGCCACGAACCACG | TTGTTGGTGGGTGAGCACAA |
| TNF | ACAACCCTCAGACGCCACAT | TCCTTTCCAGGGGAGAGAGG |

**Supplementary Table 5: List of antibody-derived tags (ADTs).**

| **Antibody-derived tags (ADTs)** |
| --- |
| CD10 |
| CD13 |
| CD15 |
| CD18 |
| CD26 |
| CD47 |
| CD69 |
| CD44 |
| CD90 |
| HLA-ABC |
| HLA-DR |
| CD117 |
| CD9 |
| CD274 (B7-H1) |
| CD273 (PDL2) |
| CD133 |
| CD34 |
| CD25 |
| IgG |
| CD24 |

**Supplementary Table 6: Medium composition for NK cell medium.**

| **Compound** | **NK cell medium** |
| --- | --- |
|  | NK MACS® Basal medium, human |
| NK MACS® Supplement | 1x |
| Penicillin/streptomycin | 100 U/ml |
| Human serum | 5% |
| IL-15 | 1 ng/ml |
