# Supplementary material for: Necroptosis triggers inflammatory interferon signatures in patient-derived metastatic breast cancer organoids
Source: Signal Transduct Target Ther. 2026 Jun 1;11:204. doi: 10.1038/s41392-026-02755-9 (PMC13226679; doi:10.1038/s41392-026-02755-9)
Supplement: Supplementary file 2 — Supplementary Data [file 41392_2026_2755_MOESM2_ESM.docx]

**Supplementary Data 1**

**
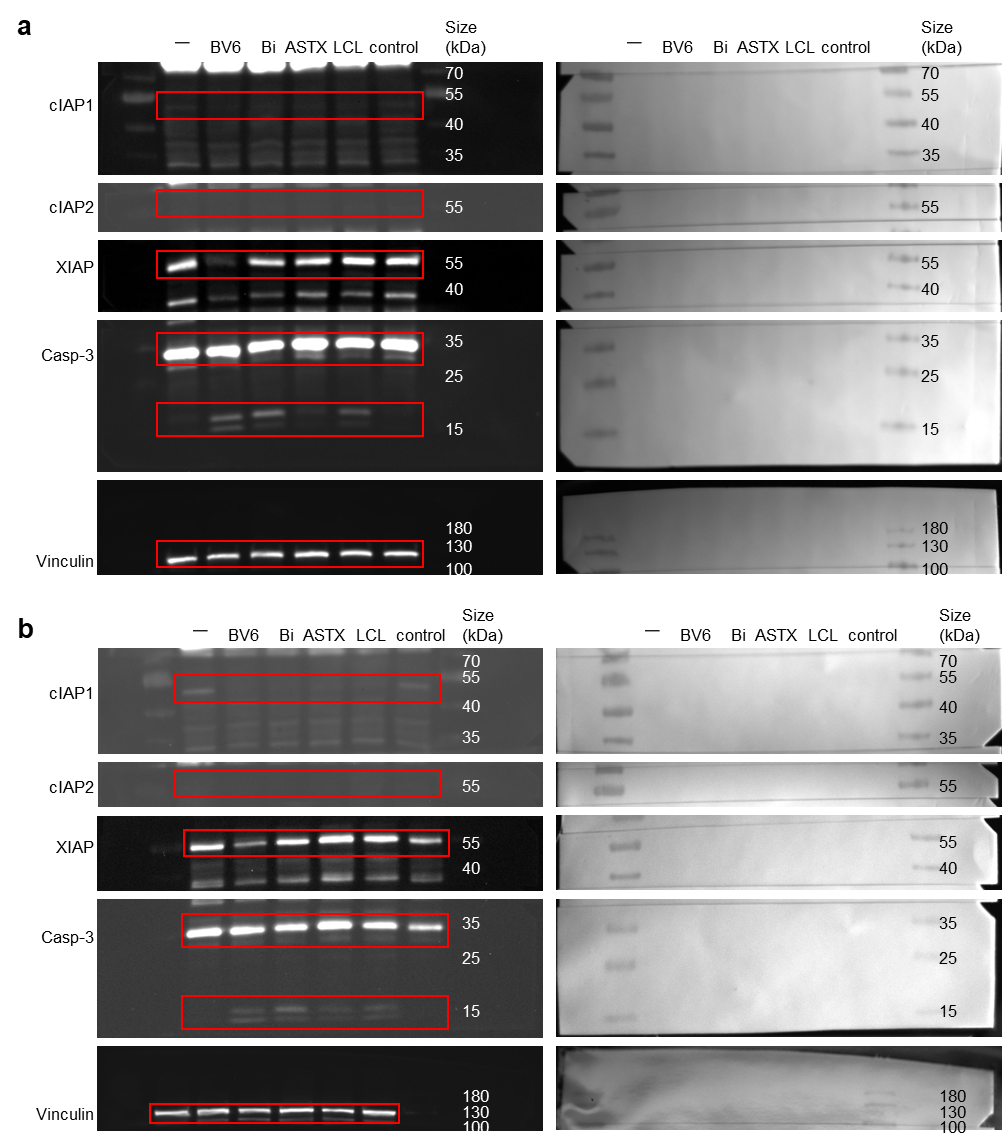
**


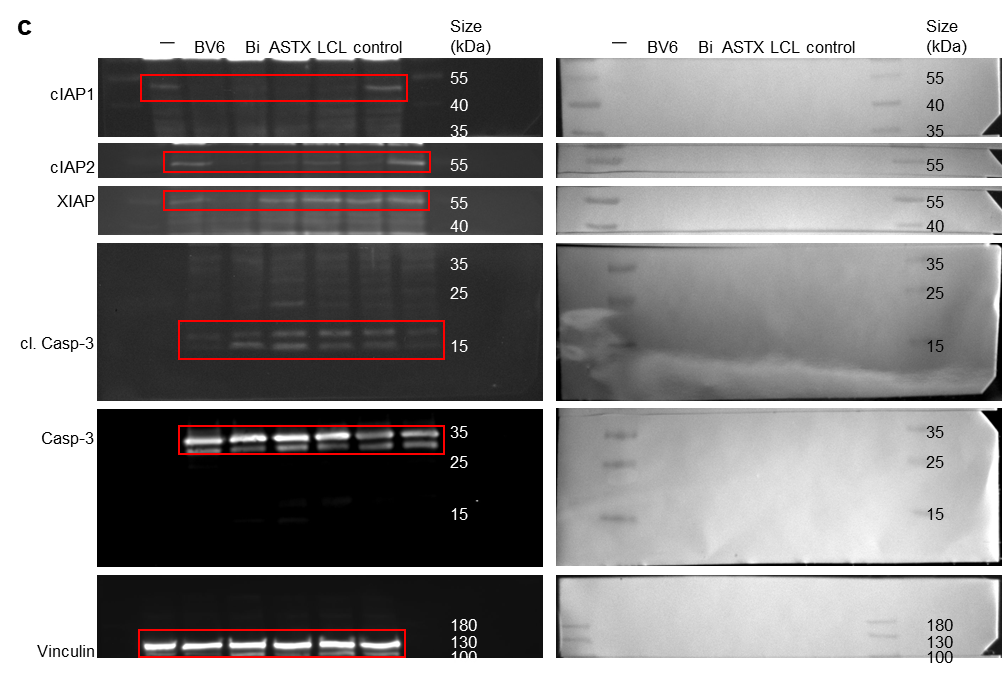


**Supplementary Data 1 corresponding to Fig. 3:** Original Western blot data from donors #1 (**a**), #2 (**b**) and #3 (**c**). The left panel shows the antibody detection and the right panel the corresponding PageRuler™ prestained protein ladder.

**Supplementary Data 2**


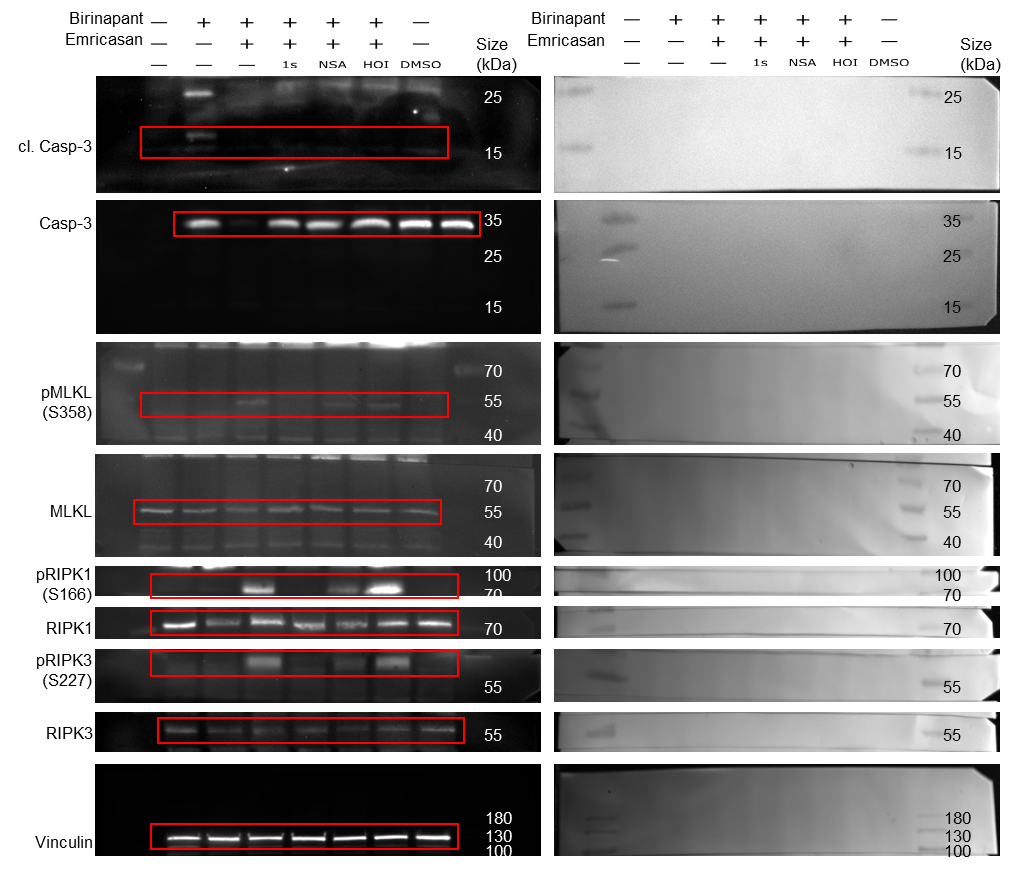


**Supplementary Data 2 corresponding to Fig. 5e:** Original Western blot data from donor #1. The left panel shows the antibody detection and the right panel the corresponding PageRuler™ prestained protein ladder.

**Supplementary Data 3**


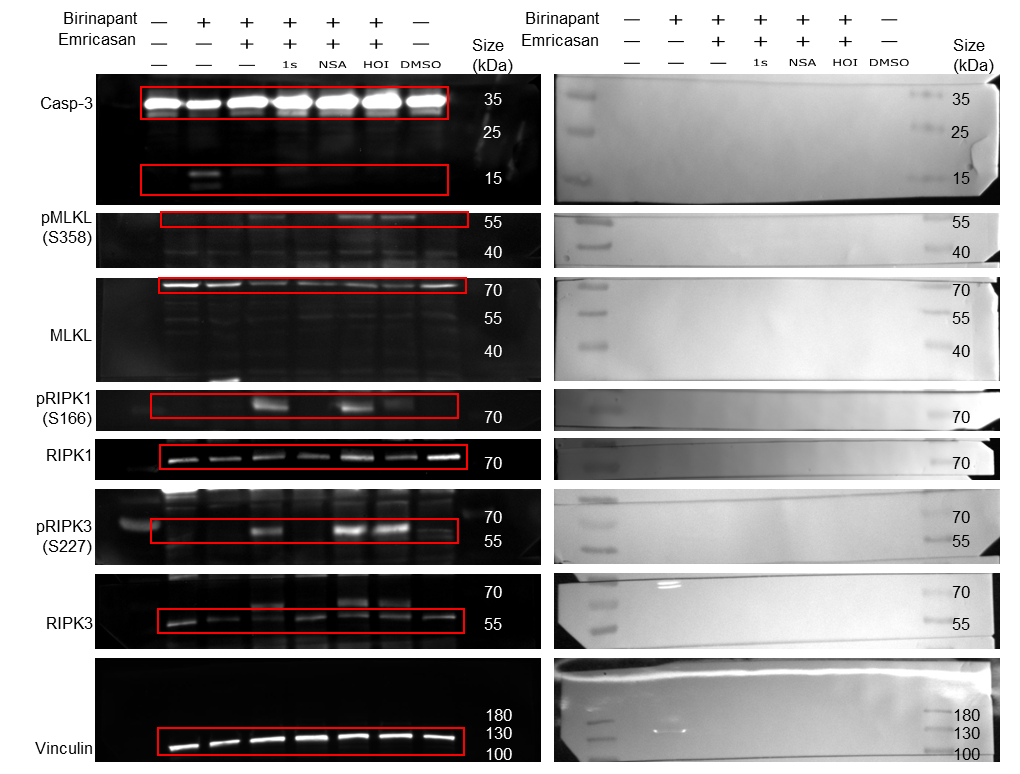


**Supplementary Data 3 corresponding to Supplementary Fig. 14e**: Original Western blot data from donor #2. The left panel shows the antibody detection and the right panel the corresponding PageRuler™ prestained protein ladder.

**Supplementary Data 4**

**
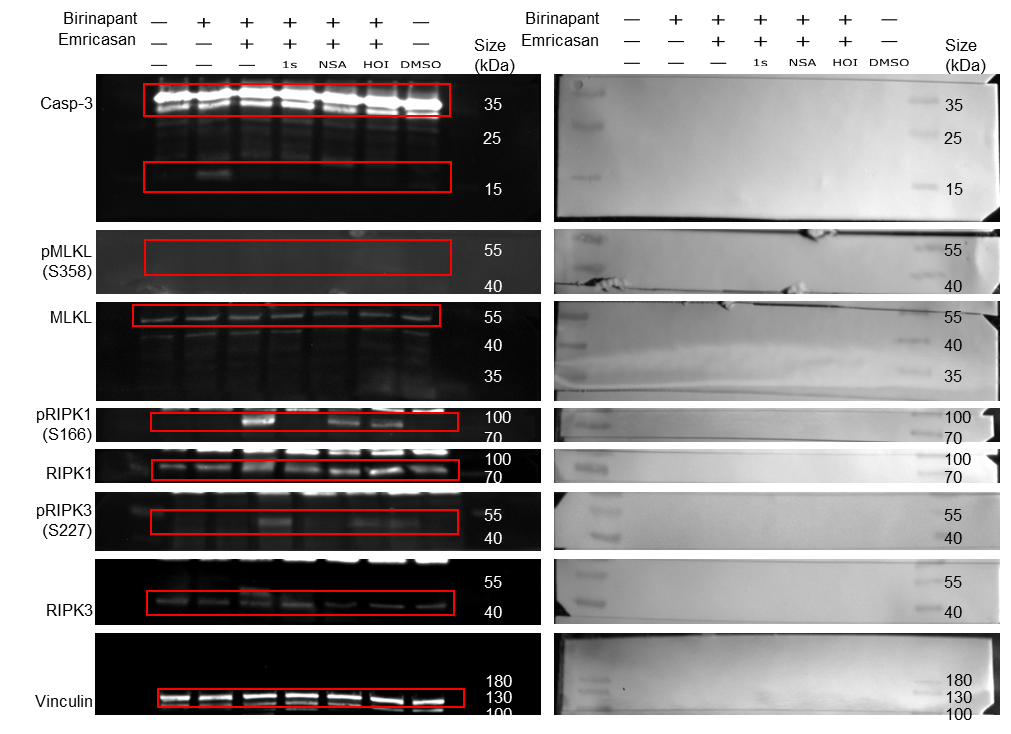
**

**Supplementary Data 4 corresponding to Supplementary Fig. 14f**: Original Western blot data from donor #3. The left panel shows the antibody detection and the right panel the corresponding PageRuler™ prestained protein ladder.

**Supplementary Data 5**

**
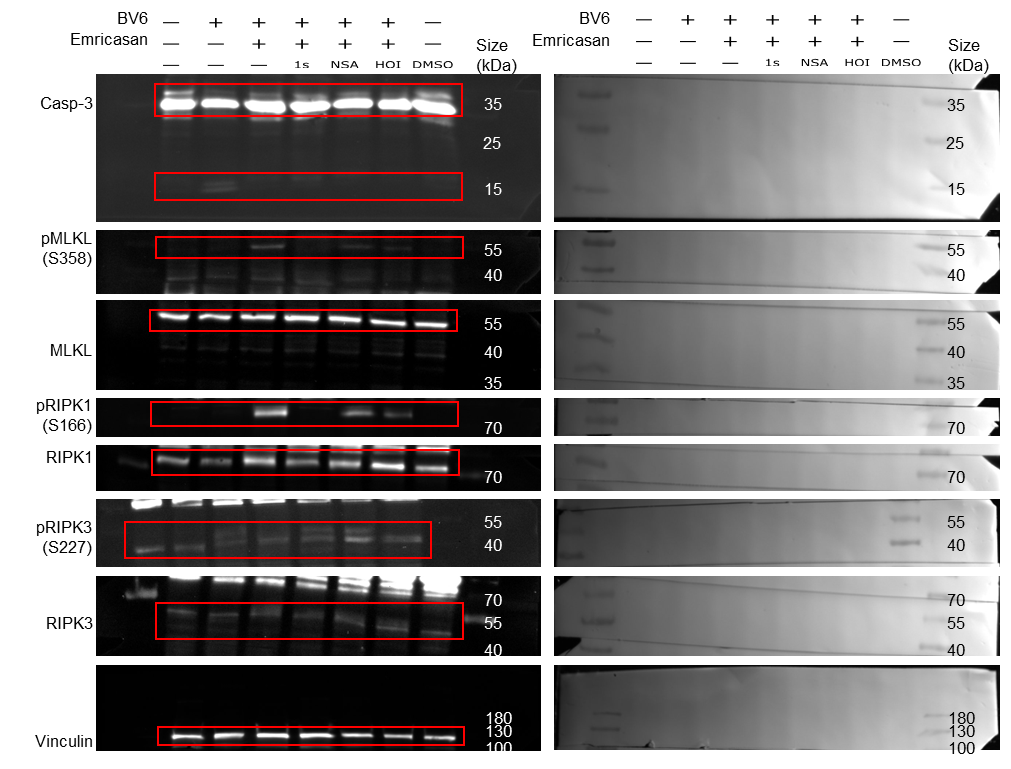
**

**Supplementary Data 5 corresponding to Supplementary Fig. 17c**: Original Western blot data from donor #1. The left panel shows the antibody detection and the right panel the corresponding PageRuler™ prestained protein ladder.

**Supplementary Data 6**

**
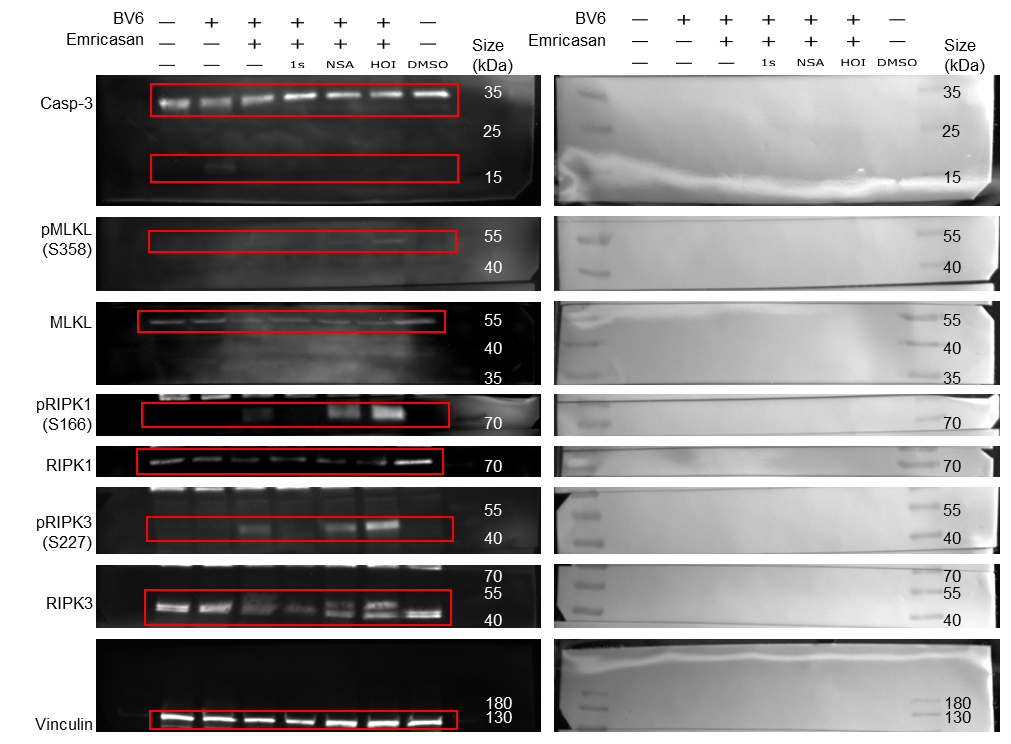
**

**Supplementary Data 6 corresponding to Supplementary Fig. 17d**: Original Western blot data from donor #2. The left panel shows the antibody detection and the right panel the corresponding PageRuler™ prestained protein ladder.

**Supplementary Data 7**

**
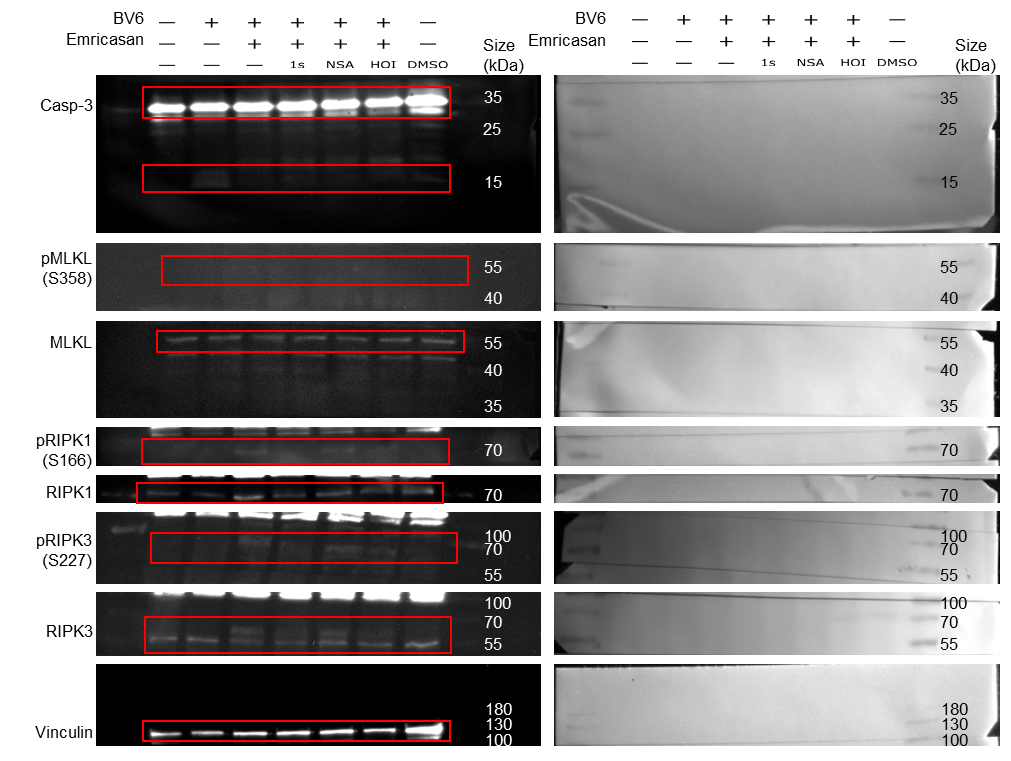
**

**Supplementary Data 7 corresponding to Supplementary Fig. 17e**: Original Western blot data from donor #3. The left panel shows the antibody detection and the right panel the corresponding PageRuler™ prestained protein ladder.
